# Pleiotropy, cooperation, and the social evolution of genetic architecture

**DOI:** 10.1371/journal.pbio.2006671

**Published:** 2018-10-25

**Authors:** Miguel dos Santos, Melanie Ghoul, Stuart A. West

**Affiliations:** Department of Zoology, University of Oxford, Oxford, United Kingdom; Massachusetts Institute of Technology, United States of America

## Abstract

Pleiotropy has been suggested as a novel mechanism for stabilising cooperation in bacteria and other microbes. The hypothesis is that linking cooperation with a trait that provides a personal (private) benefit can outweigh the cost of cooperation in situations when cooperation would not be favoured by mechanisms such as kin selection. We analysed the theoretical plausibility of this hypothesis, with analytical models and individual-based simulations. We found that (1) pleiotropy does not stabilise cooperation, unless the cooperative and private traits are linked via a genetic architecture that cannot evolve (mutational constraint); (2) if the genetic architecture is constrained in this way, then pleiotropy favours any type of trait and not especially cooperation; (3) if the genetic architecture can evolve, then pleiotropy does not favour cooperation; and (4) there are several alternative explanations for why traits may be linked, and causality can even be predicted in the opposite direction, with cooperation favouring pleiotropy. Our results suggest that pleiotropy could only explain cooperation under restrictive conditions and instead show how social evolution can shape the genetic architecture.

## Author summary

Recent research into microbial communities has revealed that the cooperative secretion of molecules—which are produced by individual cells and benefit neighbouring cells—is linked to the production of privately beneficial intracellular enzymes. This pleiotropic link between commonly and privately beneficial traits has been suggested as a novel way for maintaining cooperation in conditions under which it would otherwise be outcompeted by cheating cells. The reason is that cheats, which do not cooperate, would also lose the benefit of producing the private trait and thus suffer a fitness disadvantage. We test the plausibility of this hypothesis with analytical models and individual-based simulations. We find that cooperation can only be stabilised if one makes restrictive assumptions about the genetic architecture, such that the pleiotropic link with a private trait cannot be broken through further evolution. If the genetic architecture can evolve, then natural selection can favour mutants that do not cooperate but that still perform the private trait, leading to the breakdown of cooperation. We discuss a number of alternative explanations for the observation of linkage between cooperative and private traits and show that causality may even arise in the opposite direction to that previously predicted—when cooperation is favoured, this may select for pleiotropy. Our results suggest a novel explanation for why cooperative and private traits may be linked and show how social evolution can shape the genetic architecture.

## Introduction

Recent empirical breakthroughs in microbial genetics have suggested a novel mechanism for maintaining cooperation [1–9]. The problem with cooperation is that noncooperative cheats can avoid the cost of cooperating whilst still gaining the benefits, allowing them to outcompete cooperators [10]. Consequently, without some mechanism to favour it, cooperation would not be evolutionarily stable. In microbes, clonal growth means that interactions will often be between close relatives, and so cooperation will be favoured because it is directed towards individuals that share the genes for cooperation [10–16] (kin selection).

Experiments on both slime moulds and bacteria have led to the suggestion that pleiotropy provides a novel way to stabilise cooperation when it would not otherwise be favoured by another mechanism, such as kin selection [1–6]. These experiments have discovered cases in which two traits are either controlled by the same gene or coregulated (pleiotropy) and in which these two traits are a cooperative trait and a trait that provides a personal (private) benefit. For example, in the bacteria *Pseudomonas aeruginosa*, the quorum sensing signalling network controls both: (1) the production of several factors that are excreted from the cell and provide a cooperative benefit to the local population of cells [17–22] (public goods) and (2) private traits such as the production of intracellular enzymes involved in metabolism, and cyanide resistance [3–5]. Consequently, a mutant that did not respond to quorum sensing would avoid the cost of cooperatively producing public goods (cheats) but also pay the cost of not performing the private traits. If this cost of not performing the pleiotropically linked private goods was high enough, this could stabilise cooperation, when it would otherwise be outcompeted by cheats. In different papers, this linkage is referred to as pleiotropy, coregulation, or metabolic constraint, and it has been suggested as a key mechanism for stabilising cooperation, alongside factors such as kin selection and policing [1–9,23,24] (S1 Table).

However, these observations of pleiotropy between cooperative and private traits do not necessarily imply that pleiotropy is stabilising cooperation by preventing the invasion of cheats, as alternate explanations are possible. One possibility is that the private and cooperative traits are favoured independently, and it is just more efficient to regulate them together (pleiotropically). Another issue is the extent to which the pleiotropy hypothesis relies on the underlying genetic architecture being relatively fixed, imposing a constraint on evolution. If the genetic architecture could itself evolve, then mutation could produce individuals that still performed the other, relatively essential trait but that did not cooperate. In line with this prediction, the emergence of such cheats has been shown experimentally in the bacteria *P*. *aeruginosa*, where private and public goods are linked by quorum sensing [21]. When cooperation was not favoured, individuals rewired the control of the cooperative trait, thereby stopping its production while still producing the private trait. This resulted in a greater competitive ability relative to cooperators [18,21,25].

Another possibility is that the causal link could be in the opposite direction, with cooperation favouring pleiotropy rather than pleiotropy stabilising cooperation. For example, consider a scenario, without pleiotropy, in which cooperation was favoured by a mechanism such as kin selection. If a noncooperative mutant arose, it might initially increase in frequency locally but would eventually be outcompeted by cooperators. Nonetheless, the initial spread of these noncooperators would impose a short-term cost to the cooperators from which they arose and with which they would be competing [26–29]. Pleiotropy between cooperation and another privately beneficial trait would provide a mechanism to prevent the initial spread of cheats and hence could be favoured to reduce or avoid this ‘cheat load’ [28,30].

We theoretically model the conditions required for either pleiotropy to stabilise cooperation or the alternate possibility that cooperation favours pleiotropy. We consider a form of cooperation common in bacteria, in which cells produce a factor that is excreted from the cell and which then provides a benefit to the local population of cells [31]. This can be modelled as a public goods game, in which individuals can pay a cost to perform a task that benefits the local group. We first consider a simple scenario analytically, for which the underlying architecture is fixed and does not evolve. This analysis formalises previous verbal arguments for how pleiotropy could stabilise cooperation. We then use an individual-based simulation approach to test whether allowing the genetic architecture to evolve influences the extent to which pleiotropy is evolutionarily stable. This simulation approach also allows us to test the alternate possibility that cooperation favours pleiotropy.

## Results

### Public goods cooperation

To provide a baseline, we consider the cooperative production of public goods in a deliberately simple scenario, where there is no pleiotropy with other traits [32–35]. Our aim is to examine what happens in the simplest (null) case without recourse to details that will vary across species. We assume a haploid population subdivided into a large number of local patches, each of size *N*, where the mean genetic relatedness between individuals is *r* [36]. Individuals can secrete a public good molecule at personal cost *c*. Production of this public good provides a benefit *b* that is shared equally among all patch members, even cheats that do not contribute. We assume that this cost and benefit influences fecundity and that after reproduction, adults die, and all offspring disperse to compete globally for some new patch to start the life cycle again (nonoverlapping generations and no kin competition). The production of the public good molecule will be favoured when *rb̃* − *c̃* > 0, where *b̃* = (*N* − 1)*b*/*N*, −*c̃* = *b*/*N − c* (Eq 3 of Methods). The *b̃* term represents the average benefit that public goods production provides to the other members of the group. The −*c̃* term represents the benefit provided to the actor by their own public good production (*b*/*N*), minus the cost of producing that public good (*c*). This standard result is just Hamilton’s rule for a linear public goods game, showing how cooperation can be favoured by kin selection if the indirect benefit to relatives (*rb̃*) outweigh the direct cost (*c̃*) [10,34,37–39].

### Pleiotropic cooperation

We then examined the scenario in which the cooperative trait is linked pleiotropically to a private trait that is important for reproduction. Pleiotropic links between private and public traits have been discussed in two ways in the microbial literature, either because they are controlled by the same gene, or because they are controlled by two linked genes. For example, in *Dictyostelium discoideum*, mutants in the single gene *dim*A both do not receive the signal for the cell to differentiate into a prestalk cell and are excluded from spores [1]. In contrast, in *P*. *aeruginosa*, different genes control different traits, but these traits are linked by the quorum sensing system [2]. We consider both types and shall return to the extent to which they represent pleiotropy.

**Fig 1:**
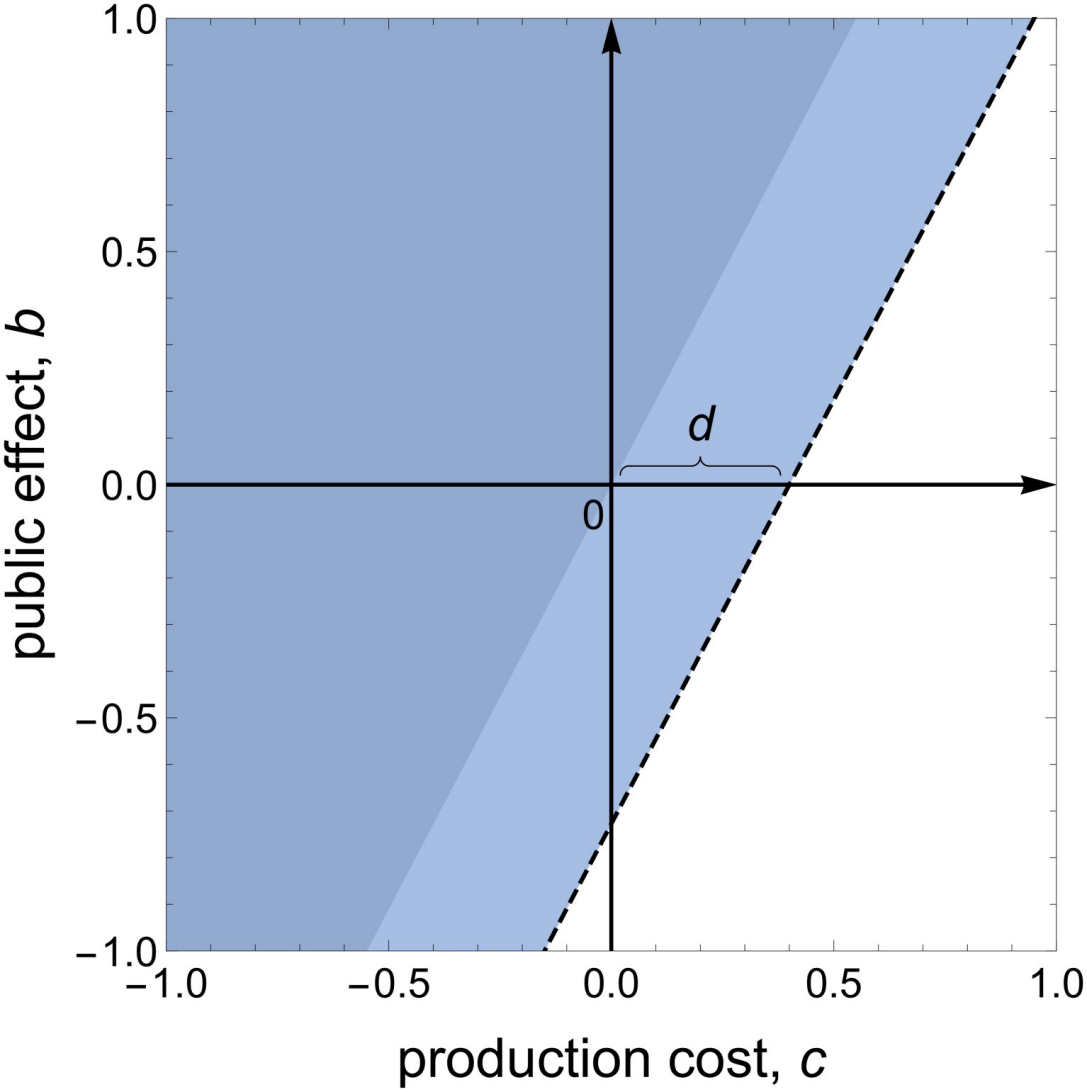
Pleiotropy can help stabilise all forms of social trait. We consider all possible forms of social interaction by considering a trait that has a fecundity effect −*c* for the individual performing it and a fecundity effect *b* that is shared amongst all the members of the group. If *b* > 0, then the trait is helpful, providing a benefit to both self and others (public good), whereas if *b* < 0, then the trait is harming and costly to both self and others. If *c* > 0, then the trait is costly to perform, but if *c* < 0, then performing the trait provides some personal benefit. The dark coloured area shows where the trait would be favoured. The area to the left of the dashed line shows where the trait will be favoured when the trait also has some pleiotropic private benefit (*d*). The lighter coloured area represents the extent to which pleiotropy can help stabilise social traits. Parameters: *N* = 10, *r* = 0.5, *d* = 0.4.

In this section, we assume that the cooperation and private traits have to be linked. This could be due to either (1) the two traits being controlled by a single gene, as in the *D*. *discoideum* example, or (2) the two traits being controlled by separate genes but the activity of these genes being linked in a way that cannot be broken by mutation (no mutational accessibility). We assume that not performing the private trait reduces an individual’s fecundity by an amount *d*. We compared the fitness of pleiotropic cooperators with individuals that lacked this pleiotropic trait and therefore neither cooperated nor performed the private trait, as has been done in laboratory experiments [2–4]. In this scenario, switching from defection to pleiotropic cooperation still changes an individual’s inclusive fitness by *rb̃* − *c̃*, but the direct fitness cost *c̃* is decreased by *d* (Eq 4). Hence, the area where cooperation is favoured is larger when cooperation is linked pleiotropically with a private trait and where that private trait provides a relative reproductive advantage (Fig 1 and S1 Fig).

An extreme case of this model is when pleiotropy links cooperation to an essential private trait. We considered this specific scenario because, in the empirical examples in which pleiotropy has linked cooperation to a private trait, the fitness of individuals that do not perform this private trait could be effectively zero [2–5]. We investigated this case by assuming that the fitness of noncooperators is zero and that the public good is equally distributed to all remaining pleiotropic cooperators. In this case, pleiotropic cooperation provides a direct fitness benefit (–*c̃* > 0), and pleiotropic cooperators always interact exclusively with other pleiotropic cooperators, and so relatedness does not matter (*b̃* = 0; Eq 5). Consequently, noncooperators are never able to outcompete pleiotropic cooperators.

### Pleiotropy promotes cooperation and anything else

Both previous verbal arguments and our above results have focused on how pleiotropy can help stabilise cooperative traits that benefit other individuals [1–8] (*b* > 0). However, our above analysis reveals that pleiotropy can also help favour traits that otherwise provide no benefits (*b* = 0, *c* > 0) or even harm (*b* < 0, *c* > 0) both their bearer and other individuals on the patch (Fig 1 and S1 Fig). Consequently, rather than pleiotropy stabilising cooperation per se, pleiotropy can help stabilise any type of trait.

For example, consider the extreme case in the opposite direction, of a harming trait that reduces the fitness of everyone in the social group, including the individual that produces it (*b <* 0). This would be analogous to bacterial cells producing an antibiotic or bacteriocin to which they are not resistant. If the genetic architecture is fixed, this harming trait would be favoured if the cost to private-good nonproducers (*d*) outweighs the sum of the cost of performing the harming trait (*c*), the cost of harming relatives ([*N*– 1]*b*/*N*), and the cost of harming oneself (*b*/*N*). An analogous point has been made in the cooperation in human literature, in which it was pointed out that punishment could help stabilise anything and not just cooperation [40].

### The evolution of genetic architecture

Our above analyses have assumed that pleiotropy is a fixed constraint on the genetic architecture, such that individuals can either perform both the private and the public trait or perform neither. This could occur via the two traits being controlled by a single gene or by two linked genes whose link cannot be broken by mutation. However, the genetic architecture can evolve over time [21,41–43], and assuming that it cannot evolve could result in misleading conclusions [44,45]. For example, there is no reason that the private traits that have been observed to be pleiotropically linked to cooperation in bacteria could not in principle be independently regulated [21,46,47]. The key point here is that mutational accessibility, or pathway rewiring, could break the pleiotropic link, making traits independent. Previous verbal arguments and our above model have assumed that neither mutation nor different expression pathways can unlink the cooperation and private traits.

We used an individual-based simulation approach to investigate the consequences for cooperation of allowing mutation to unlink the cooperation and private traits and hence allow the genetic architecture to evolve. To approximate a microbial life history, we assumed a haploid population in which subpopulations grow with clonal reproduction on patches before dispersing to compete globally for new patches. Because reproduction is clonal, both traits are perfectly transmitted to daughter cells. The population is subdivided into *n*_*p*_ = 100 patches. At the beginning of the life cycle, each patch is colonised by *N*_*F*_ = 100 founders, which leave *N* = 10 initial individuals on the patch. In order to vary relatedness across the entire range from *r* = 0 to *r* = 1, we vary the likelihood with which these 10 initial individuals are sampled from the same parent founder. This allows us to vary relatedness without varying the number of initial cells, as would occur when different numbers of lineages colonise a patch and can then grow clonally [48]. The cells on a patch potentially produce a public good and grow for *k* = 10 generations. We assume that individuals have a baseline growth rate of 1 + *g* so that population size grows. Mutations occur during the growth phase. During the 10 generations of growth, only parents die after each reproduction event, and therefore, there is no population regulation, and generations are nonoverlapping. After these 10 generations, the remaining cells disperse globally and begin the life cycle again.

**Fig 2:**
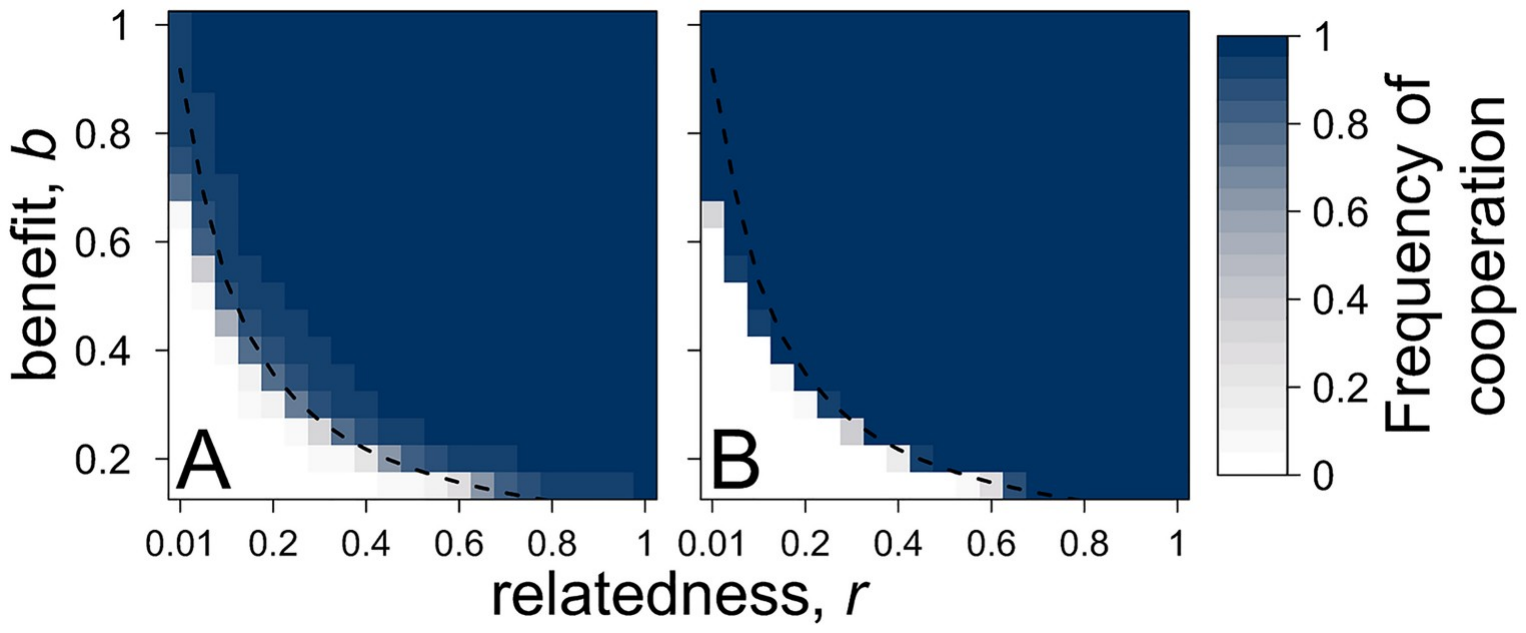
If the genetic architecture can evolve, pleiotropy does not stabilise cooperation. (A) Nonpleiotropic cooperators compete with cheats. (B) A pleiotropic link is allowed to coevolve with public good production (all genotypes and mutations I and II in Fig 3). The *x* axis is the relatedness, *r*, measured between the *N* individuals after patch colonisation. The dashed line represents the analytical prediction, *c* − *b*/*N* = *r*(*N* − 1)*b*/*N*, for when Hamilton’s rule is satisfied, assuming that migration occurs every generation (i.e., *k* = 1; Eq 3 in the Methods section). The frequency of cooperation was measured as the proportion of cooperative genotypes in the population (i.e., pleiotropic and nonpleiotropic cooperators and cooperative private nonproducers; Fig 3). Irrespective of whether pleiotropy can evolve, the cooperative production of public goods is only favoured when Hamilton’s rule is satisfied (*rb̃ − c̃* > 0). Parameters: *c* = 0.1, *g* = 0.5, growth phase *k* = 10, mutation rate *μ* = 0.001.

For comparison, we first analysed the scenario in which there is no pleiotropic private trait. Our simulation results for this case were in close agreement with our analytical predictions—cooperation is favoured as predicted by Hamilton’s rule (Fig 2A). Cooperation in our simulation evolves under a slightly larger area than predicted by our analytical model, because relatedness increases during the growth phase, especially at low initial relatedness (see top-left corner of Fig 2A and 2B and S2 Fig).

We then considered the scenario in which cooperation is always pleiotropically linked to a private trait and in the specific case when not producing the private good leads to the individual not reproducing (i.e., their fecundity is 0). In this case, the only possible mutation is to not express both traits, as has been assumed previously [1–9,23]. We focused on this extreme scenario, as a conservative assumption, because it is the case in which pleiotropy could be most effective in stabilising cooperation. Again, our simulation results were in agreement with our analytical results—pleiotropic cooperation is always favoured over noncooperators regardless of relatedness, but the population goes extinct when the average fecundity is lower than 1 (S3 Fig). In the supplementary information, we relaxed the assumption that private nonproducers have fecundity 0 and let them reproduce, albeit with a baseline fitness that is reduced by *d*. We show that pleiotropic cooperation prevails as long as the cost of not expressing the private trait *d* is sufficiently high (S4 Fig).

**Fig 3:**
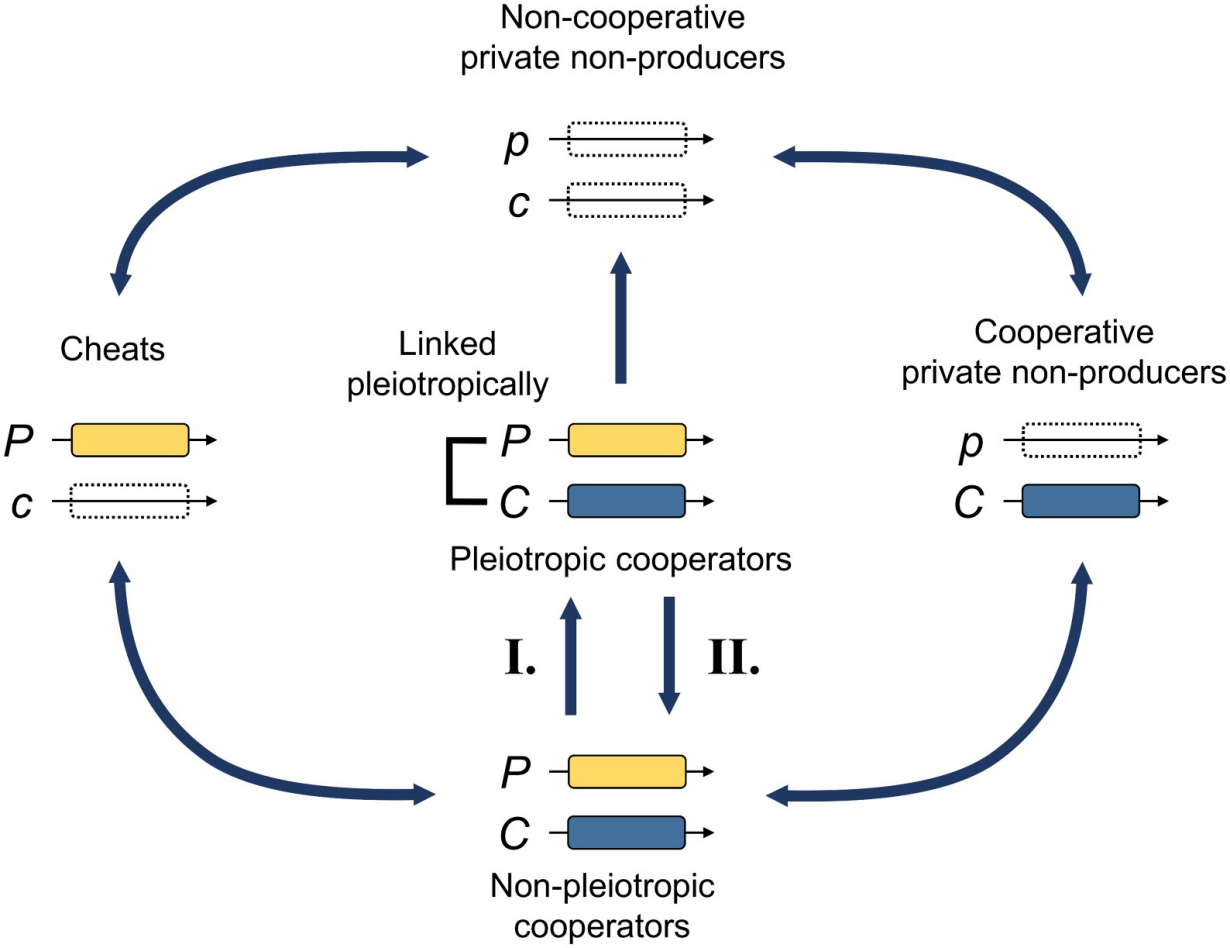
Genotypes studied in our model and the possible mutational pathways (blue arrows). Pleiotropy can evolve from a genetic architecture in which the private trait (*p*, *P*) and cooperation (*c*, *C*) are unlinked (mutation I) and is either allowed or not allowed to revert (mutation II). All other mutations are always possible. During reproduction, a mutation occurs with probability *μ* in each offspring, in which case either one randomly chosen locus or the pleiotropic link in cooperators is changed. We also performed simulations with more realistic mutation rates, for which the probability of gaining a function (either cooperation, the private trait, or pleiotropy) was reduced by a factor of either 10 or 100 (S5 and S6 Figs).

We then examined the consequences of allowing the genetic architecture to evolve (Fig 3). We allowed mutation from a genotype in which both the private and public goods are linked pleiotropically (pleiotropic cooperators) to one in which they are independent (nonpleiotropic cooperators) and vice versa. Hence, pleiotropy can be acquired or lost (mutations I and II in Fig 3). Pleiotropic and nonpleiotropic cooperators are otherwise phenotypically similar. As a consequence, pleiotropy is now also in competition with all nonpleiotropic genotypes, for which the private and public good traits are independent (Fig 3). Once traits are independently regulated, a single mutation will influence only one of the traits and not both, as the link between them has been broken. As above, we consider the extreme case in which the private trait is essential, such that individuals that do not produce it cannot reproduce.

When we allowed the genetic architecture to evolve, we found that the potential for pleiotropy, where the traits could be linked, did not favour cooperation. Specifically, the production of public goods was only favoured in the same area as when there was no potential for pleiotropy (compare Fig 2A and 2B), which is when the indirect benefits outweighed the direct costs, and Hamilton’s rule was satisfied (*rb̃ − c̃* > 0). In the other areas, where public good cooperation was not favoured by kin selection, mutation can create individuals for which the private trait and public goods production are not linked (mutation II in Fig 3). These individuals can then mutate to create a cheat that does not produce the public good but still performs the private trait. Selection favours these cheats, which can then invade and outcompete pleiotropic cooperators.

Our result shows that in a simple case, if the underlying genetic architecture can evolve, then pleiotropy does not provide a mechanism to stabilise cooperation. Our aim was to capture the key feature of the ‘pleiotropy stabilises cooperation’ hypothesis, that private and public traits could be linked. Consequently, we assumed that the linkage between traits could either be gained or lost by mutation, which could be applied to a range of specific genetic architectures.

More generally, our results illustrate how assuming fixed genetic associations between cooperation and other traits can lead to misleading conclusions. The problem is that assuming certain types of associations can force evolutionary outcomes that would otherwise be unlikely. This has been discussed previously in the context of traits such as punishment, rewarding, kin recognition, and green beards [45,49–52]. Furthermore, our conclusions are supported by a recent experiment in *P*. *aeruginosa* that showed the evolution of cheats that circumvent the expression of a cooperative trait but still produce a private trait [21].

### Robustness and different genetic architectures

We tested the robustness of our results by relaxing our assumption about how mutation can influence the genetic architecture (mutational accessibility) in three ways. In all three cases, we found essentially identical results, that the potential for pleiotropic links between traits did not stabilise cooperation. First, we ran simulations with more realistic mutation rates, in which losing a function was either 10 or 100 times more likely than gaining a function (S5 and S6 Figs). Second, we allowed the genetic architecture to evolve in a number of different ways, by either preventing pleiotropy to be lost, allowing pleiotropic cooperators to generate viable cheats, or allowing all mutations (section S1.1 of S1 Text and S7 Fig). Third, we explicitly modelled the link between the cooperation and private traits by introducing a regulator for each trait and allowing the private trait’s regulator to become universal (pleiotropic) and hence regulate both traits at the same time (section S1.2 and S8–S11 Figs).

In contrast, Frénoy and colleagues [6] suggested that the potential for genetic links between private and cooperation traits can help populations resist the invasion of cheats. They found that where there was more pleiotropy between cooperative and metabolic traits, cooperation was lost more slowly and in some cases was maintained and that when allowing cooperation to evolve de novo, it evolved with links with metabolic traits. Our models differ in a number of ways, and therefore, we cannot say for sure why our conclusions differ. One possibility is that, because some of the pleiotropic interactions by Frénoy and colleagues [6] were due to gene overlap and/or operon sharing, the pleiotropic link could not be easily broken (personal communication, D. Misevic). The resistance of such pleiotropy to cheat invasion could have been influenced by a number of factors. For example, (1) the values chosen (analogous to a single combination of *b* and *r*) may have led to weak selection against cooperation and hence weak selection for unlinked genetic architecture; or (2) maybe longer simulations (>2,000 generations) or larger population sizes (>1,024 individuals) were required for the combination of mutation and selection to break up the links. Targeted simulations of different scenarios would allow the importance of these issues to be determined.

### Cooperation promotes pleiotropy

Our above results suggest that if the genetic architecture can evolve, pleiotropy will not help stabilise cooperation (Fig 2). Instead, the private and public good traits have to be linked in such a way that the pleiotropy is unavoidable for pleiotropy to stabilise cooperation (Fig 1; S1 and S3 Figs). Considering the empirical examples from microbes, it is not clear why the traits would always have to be linked pleiotropically [1–6,46,47]. For example, the production of intracellular and extracellular factors in bacteria could evolve to be controlled by different circuits [21]. We suggest that unless it is explicitly shown otherwise, the null hypothesis should be that the genetic architecture can evolve, and hence, pleiotropy will not help stabilise cooperation.

**Fig 4:**
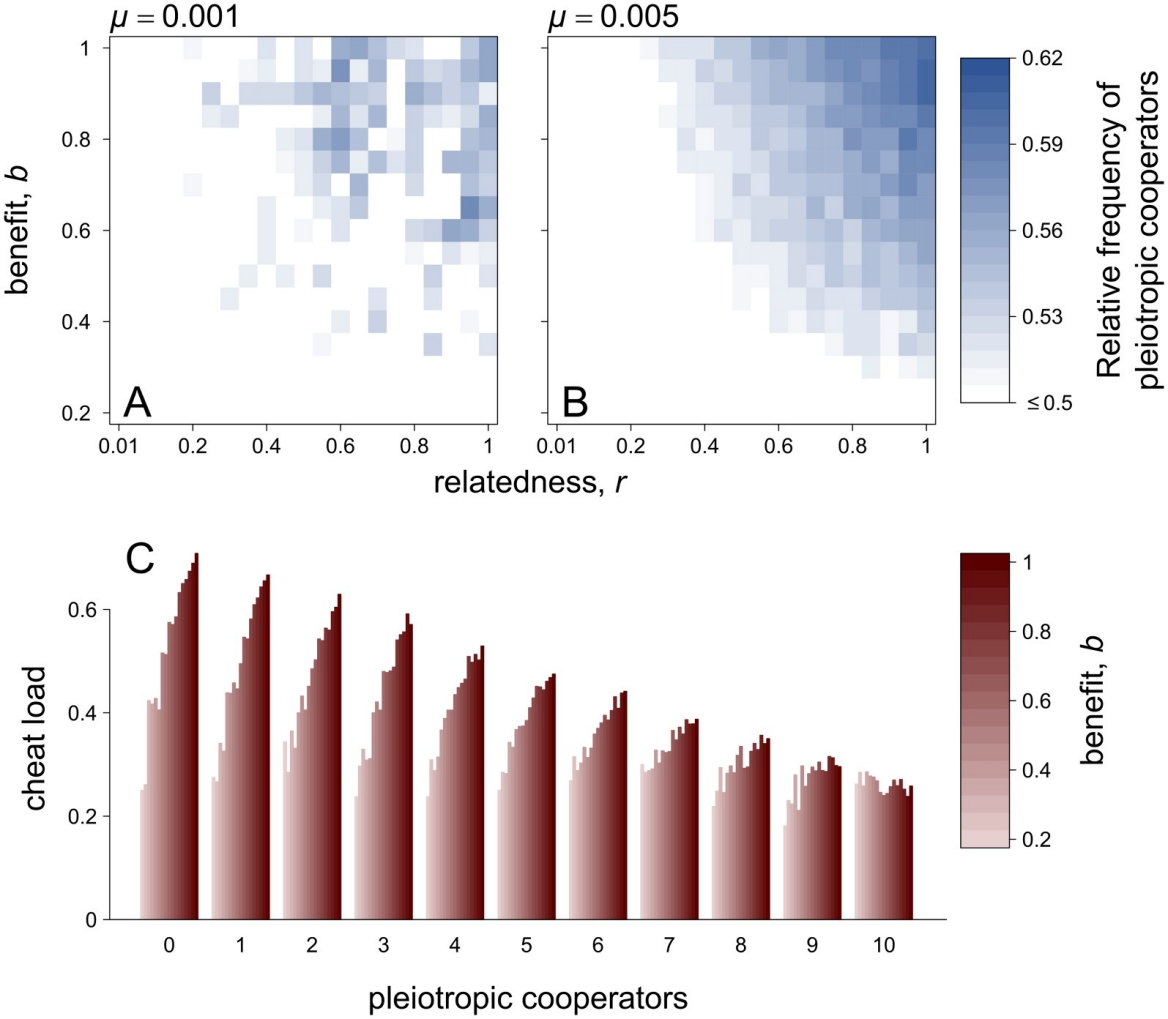
Cooperation promotes pleiotropy. (A) and (B) show the proportion of pleiotropy relative to all cooperative genotypes (i.e., pleiotropic cooperators, nonpleiotropic cooperators, and cooperative private nonproducers). In both panels, when both relatedness (*r*) and the benefit of cooperation (*b*) are high, and thus there is strong selection for cooperation, pleiotropy is favoured. Because pleiotropic and nonpleiotropic are phenotypically similar, we expect them to be present in similar proportions relative to each other in the absence of mutations (i.e., 0.5). Hence, a frequency of pleiotropic relative to nonpleiotropic cooperators above 0.5 shows evidence of an advantage to pleiotropic cooperators. A comparison of panels (A) and (B) shows that increasing mutation rate *μ* leads to more pleiotropy. Panel (C) shows the cheat load on local patches during a single growth phase (mutation rate *μ* = 0.005) for different values of the cooperation benefit *b*. Each patch is started with 10 cooperators, and the *x* axis shows the number of those cooperators for which cooperation was pleiotropically linked to an essential private trait. Cheat load is computed as follows. We measure the difference between average fitness without mutations, *W*_*max*_ (maximum attainable average fitness for cooperators in the absence of cheats), and average fitness with mutations, *W* [28,30]. The bars show this difference expressed as a percentage of maximum attainable fitness, i.e., 100 × (*W*_*max*_ *− W*)/*W*_*max*_. Increased pleiotropy leads to a decreased accumulation of cheats and, in turn, a lower cheat load. Both *W*_*max*_ and *W* are averages taken over 10^4^ patches. In all panels, all genotypes and mutations I and II in Fig 3 are allowed. Parameters: *c* = 0.1, *g* = 0.5, *k* = 10.

We hypothesise that causality could even be in the opposite direction, with cooperation favouring pleiotropy. We tested this by comparing the relative proportion of pleiotropic and nonpleiotropic cooperators in the area where Hamilton’s rule was satisfied. We examined the proportion of pleiotropic cooperators relative to nonpleiotropic cooperators—rather than relative to all nonpleiotropic genotypes (including cheats)—for the following reason. When Hamilton’s rule is satisfied, both pleiotropic and nonpleiotropic cooperators are fully favoured (Fig 2B). In this case, the null hypothesis is that they are equally common (i.e., 0.5). A proportion of pleiotropic cooperators relative to all cooperative genotypes above 0.5 would show evidence that pleiotropy is indeed favoured. In support of our hypothesis, we found that, when Hamilton’s rule was satisfied, pleiotropic cooperators were relatively more common than nonpleiotropic cooperators, provided both the public good benefit (*b*) and relatedness (*r*) were high (Fig 4A and 4B). Consequently, pleiotropy is being favoured in the conditions under which cooperation is strongly selected for by kin selection (Fig 4A and 4B). We found similar results when the pleiotropic link was modelled more explicitly (S5 and S6 Figs). A similar point that genetic assortment (i.e., relatedness) could favour pleiotropy was hypothesised by Frénoy and colleagues [6], but they did not test whether increasing assortment, and hence selection for cooperation, leads to more pleiotropy.

**Fig 5:**
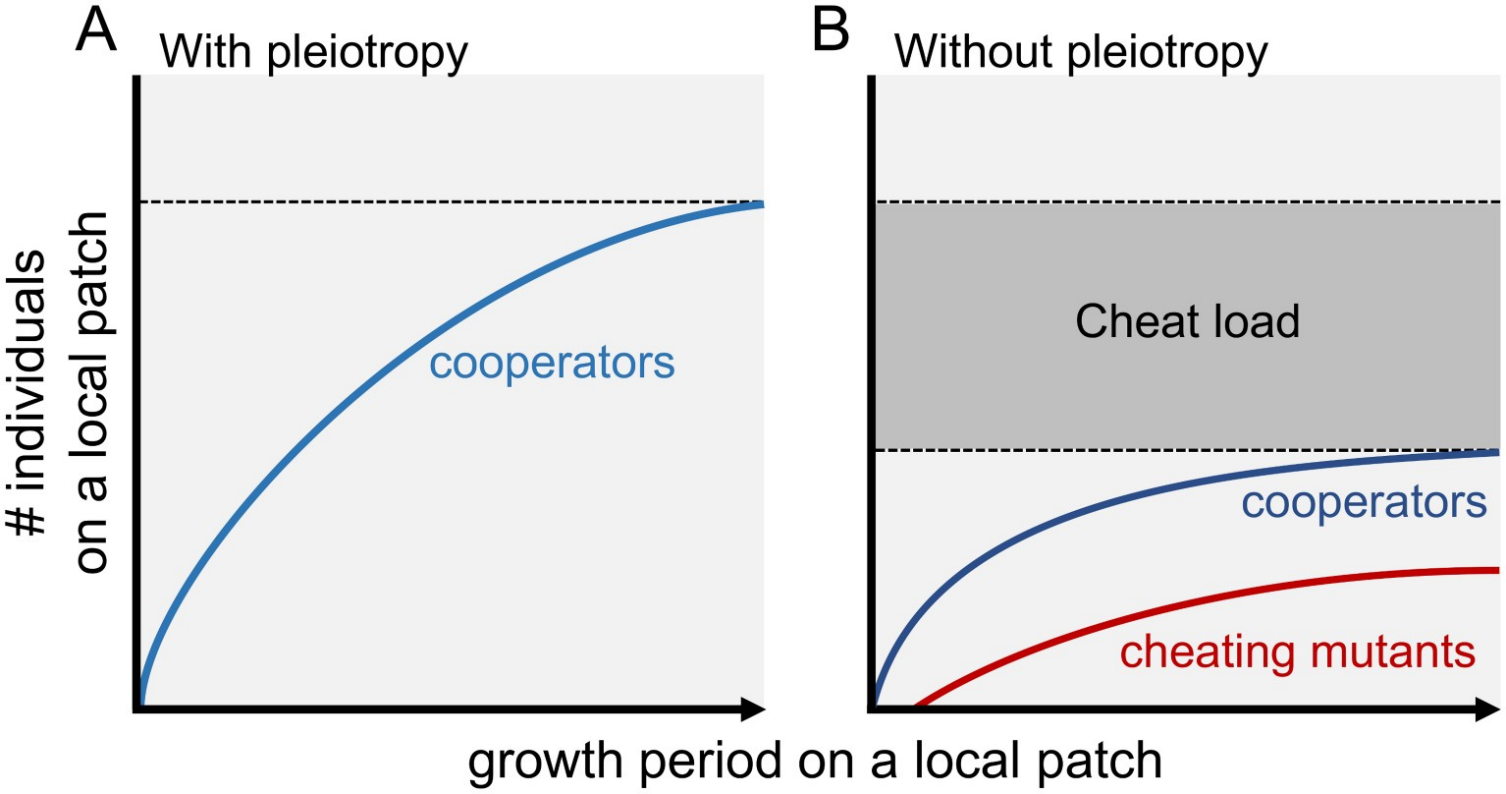
The cheat load hypothesis. (A) When cooperation is favoured, pleiotropic cooperators that undergo mutations generate cheating mutants that do not express an essential private trait and thus are not able to grow locally. (B) When cooperation is not linked to an essential private trait, noncooperators (cheats) are able to grow locally, which reduces the absolute number of dispersing cooperators. Patches with more pleiotropic cooperators export more individuals, and therefore, cooperators with pleiotropy have an advantage over cooperators without pleiotropy. Pleiotropy does not influence the extent to which cooperators are stable against the invasion of noncooperative cheats in the long term.

In our model, cooperation favours pleiotropy because it reduces the mutational ‘cheat load’ locally (Fig 5). If the production of public goods is favoured by kin selection, then any nonproducers that arise by mutation will eventually be outcompeted [32,34]. However, nonproducers will be able to initially invade within a subpopulation [18,19,53–57]. This initial invasion will reduce the fitness of the cooperative lineage from which they evolved [27–29]. Pleiotropic linkage with a private trait prevents the nonproducers from invading and hence provides a fitness advantage to pleiotropic cooperative lineages, relative to cooperative lineages where production of the public good is not linked to a private trait. The key point here is that pleiotropy is only favoured if cooperation was otherwise favoured by a mechanism such as kin selection. Consistent with this cheat load hypothesis, an increase in the mutation rate makes pleiotropy more likely to be favoured (Fig 4A and 4B).

To test whether nonpleiotropic cooperative lineages did suffer a cheat load, we ran additional simulations to examine how pleiotropy influenced growth in patches of cooperators. We compared the cheat load at the end of a single growth phase between conditions with and without mutations in patches started with 10 cooperators but where there were *N*_*PC*_ pleiotropic cooperators and 10 − *N*_*PC*_ nonpleiotropic cooperators. Without mutations, patches can grow maximally and reach maximum average fitness *W*_*max*_ = 1 + *g* − c + *b*/*n* + (*n* − 1)*b*/*n* = 1 + *g* − c + *b*, as no cheat can emerge. With mutations, however, cheats emerge and impair their local patch growth. Cheat load was therefore measured as the difference between the maximum attainable average fitness in the absence of mutations and the observed average fitness with mutations *W* [28,30]. As predicted, patches with more pleiotropic cooperators at the beginning of the growth phase eventually suffered a lower cheat load and thus had a higher average fitness than patches with more nonpleiotropic cooperators (Fig 4C). The reason is that it takes more mutations to generate viable cheats from a genetic architecture in which private and cooperation traits are pleiotropically linked, as in [6]. In pleiotropic cooperators, the private and cooperation traits first have to be unlinked. This generates nonpleiotropic cooperators, which in turn need to undergo a second mutation to generate viable cheats (Fig 3).

Our results emphasise the importance of distinguishing between short-term invasion and long-term evolutionary stability. Pleiotropy does not help stabilise cooperation over evolutionary time—cooperation is only favoured in the region where Hamilton’s rule is satisfied because of indirect fitness benefits (Fig 2). In contrast, pleiotropy does slow down the extent to which cheats will arise and invade in the short term (Fig 4C). The crucial point is that these cheats would not be successful in the long term—as soon as they disperse to a new patch, their fitness would be effectively zero. What matters for evolution is the long-term dynamics. Consequently, although pleiotropy protects cooperators against the short-term invasion of cheats, it does not influence whether cheats can successfully invade and outcompete cooperation on an evolutionary timescale. Another way of thinking about this is that pleiotropy provides an evolutionary advantage against nonpleiotropic cooperators but not against noncooperative cheats (Fig 5).

The extent to which pleiotropy is favoured will depend upon a number of factors. Pleiotropic and nonpleiotropic cooperators are phenotypically similar, and the cheat load is generated from rare mutations. Consequently, the evolutionary forces favouring pleiotropic over nonpleiotropic cooperators are actually weak, explaining why pleiotropy does not go to fixation (Fig 4). We found similar results when mutations were 10 times more likely to lead to a loss rather than a gain of function. However, pleiotropy was favoured only with high relatedness and large cooperation benefits, even more so with a mutation bias of 100 (in which case both *r* and *b* need to be close to 1 for pleiotropy to be favoured with *μ* = 0.005; S6 Fig). More generally, our argument is analogous to how pleiotropic mechanisms could help control the spreads of cancer, if cancer can be conceptualised of as a kind of cheat, within its multicellular host [58,59]. Another issue is that if private traits were not essential, then mutation could generate viable—albeit less fit—cheats that also do not produce private traits. Here, patches with pleiotropic cooperators would still suffer less from cheat load than patches with nonpleiotropic cooperators, where cheats that produce the private would be generated by mutations. In this case, we predict that the cheat load effect would still favour pleiotropy, though to a lesser extent than in the extreme case in which the private trait is essential.

In contrast to how pleiotropy can favour any type of trait (Fig 1), our above argument that cooperation favours pleiotropy only works for cooperative traits. A privately beneficial trait would not favour pleiotropy, because the loss of that trait would incur an immediate fitness cost anyway. Also, mutants in either privately beneficial traits do not impose a growth cost locally, because there are no social interactions. There are no cheats if the two traits are private. In contrast, even though they are selected against in the long term, cheats gain a short-term advantage, which is costly to the cooperators they are invading—pleiotropy is favoured to remove this short-term advantage. In confirmation of this, when we examined a privately beneficial trait (*b* = 0 and *c* < 0) in our simulations, rather than a cooperative trait, we found that pleiotropy was not favoured (S12 Fig).

### Fluctuating environments

We further hypothesised that the cheat load problem could be exacerbated in fluctuating environments, where cooperation alternates between being favoured and not favoured. The reason is as follows. If cooperation becomes unfavourable, cheating becomes strongly selected for. Then, local patches with more pleiotropic cooperators, which generate fewer cheats, would produce a higher number of cooperators and hence have a higher fitness when the conditions favouring cooperation returned. To test this idea, we ran simulations in which every 10 generations (i.e., every growth phase), cooperation alternates between being beneficial (*b* > 0) and not beneficial (*b* = 0).

We found that fluctuating benefits *b* decrease the region where cooperation is favoured (S13A and S13C Fig). This is because the benefit *b* = 0 half the time. As before, we found that cooperation was only favoured in the region where Hamilton’s rule was satisfied and hence favoured by kin selection (*rb̃ − c̃* > 0; S13 Fig). Furthermore, and also as before, pleiotropic cooperators were increasingly common when benefits are large and *r* is high and hence when cooperation was otherwise favoured (S13B and S13D Fig). Comparing simulations with and without pleiotropy, we found that when pleiotropy was allowed to evolve, it decreased the extent to which cheats built up and hence reduced the ‘cheat load’ (S14 Fig). We found qualitatively similar results with fluctuating population structure (alternating between *r* = 0.01 and *r* > 0.01), although cooperation decreased to a lesser extent (S15 and S16 Figs). This further confirms that if cooperation is first favoured because of kin selection, pleiotropy does not stabilise cooperation after a subsequent decease in relatedness. Overall, these results in fluctuating environments provide further support to how cooperation could favour pleiotropy.

### Mutations in pleiotropically regulated genes

In the previous sections, we made the restrictive assumption that mutations cannot occur in pleiotropically regulated genes. For example, when pleiotropy was acquired, no mutations could knock out either the private or public traits: mutants either lost pleiotropy or stopped expressing both genes. In experiments, however, mutants that lose the ability to express the public trait but keep expressing the essential private trait do exist [2]. Such mutations are expected to reduce the advantage of pleiotropy because they generate viable, private good–producing cheats.

To test how mutations in pleiotropic genotypes influence the resistance of pleiotropy against cheat load, we ran simulations in which we allowed mutations at both the private and public good loci in pleiotropic individuals, thereby varying mutational accessibility. As before, we found that pleiotropy is favoured when cooperation provides large benefits and relatedness is high, especially when the mutation rate is also high (S17A and S17B Fig). Although these simulations led to a higher frequency of cheating mutants (compare Fig 4C and S17C Fig), patches with more pleiotropic cooperators still produced fewer cheating mutants (S17C Fig). This is because a fraction of the mutations in pleiotropic cooperators generate noncooperative private nonproducers that cannot profit from public goods and hence do not impair their local patch growth. Overall, these results confirm that cooperation promotes pleiotropy rather than the reverse, even under these relaxed conditions.

More generally, we have taken a relatively heuristic approach, but there are many possible ways to model genome circuits that could lead to pleiotropy. Genetic details could influence the extent to which pleiotropy reduces the cheat load and hence the extent to which it is favoured, as well as the time required for mutations to break this pleiotropic link. For example, introducing a mutation bias in our simulations led to a considerably reduced advantage of pleiotropy against cheat load. Pleiotropy was favoured only with both very high relatedness and large cooperation benefits (S5 and S6 Figs). Consequently, as we find out more about specificities, it could be useful to model other genetic circuits.

## Discussion

### What now?

Overall, there are at least four scenarios that could explain the observed instances of pleiotropy between a private and a cooperative trait [1–5]: (1) the association between the private and cooperative traits is a relative coincidence, with no adaptive significance; (2) both traits are favoured under the same conditions, and there is an efficiency benefit to having them coregulated; (3) pleiotropy stabilises cooperation; or (4) cooperation favours pleiotropy. The first hypothesis is our null model. The second hypothesis is analogous to a common explanation for why multiple traits are controlled by quorum sensing, with different traits being favoured at different stages of the growth cycle and at different population densities [25,60–62]. Our theoretical results show how the fourth hypothesis could work and that the third hypothesis requires extremely restrictive assumptions, making it unlikely to be generally important. The critical question for future work is how these different hypotheses could be distinguished empirically.

The second hypothesis, that both private and cooperation traits are favoured under the same conditions and that there is an efficiency benefit to having them coregulated, does not require any particularly restrictive assumptions and fits with what we know about traits such as quorum sensing [25,60,61,63]. We therefore suggest that it is the most likely explanation for the observed instances of pleiotropy between a private and a cooperative trait in bacteria [1–5].

The third hypothesis, that pleiotropy stabilises cooperation, requires two patterns, neither of which have been supported by the empirical data. First, we have shown that pleiotropy only helps stabilise cooperation in conditions where cheats would otherwise invade, if there is some constraint that forces the personal and cooperative traits to be linked, such that they could not conceivably be regulated independently (i.e., the link cannot be broken by mutation). Considering the empirical examples in bacteria, there seems to be no reason that traits such as cooperative extracellular proteases and private adenosine metabolism could not be regulated independently [3]. Cheats that stopped producing the cooperative trait by rewiring its control have actually been shown to emerge and invade cooperative populations [21]. In contrast, in the *D*. *discoideum* example, the cooperation and private traits appear to be controlled by the same gene, and so it is more plausible that they have to be linked [1].

The second pattern required to support the pleiotropy favours cooperation hypothesis is that that cooperation was not stabilised by some other factor, such as kin selection. In the slime mould *D*. *discoideum*, the average relatedness in fruiting bodies is *r* = 0.98 [14], and both experimental evolution and genomic analyses also support a role of kin selection in favouring cooperation [16,64,65]. In the bacterium *P*. *aeruginosa*, we do not know relatedness in nature, but clonal growth in spatially structured populations is likely to lead to a high relatedness [11,48,66–68]. Consequently, there is no evidence that the examples of pleiotropy are in species in which cooperation is not explained by kin selection.

In contrast, the fourth hypothesis, that cooperation promotes pleiotropy, makes the opposite predictions. It does not matter if the traits could be regulated independently, and pleiotropy is more likely to be observed in cases in which relatedness is high; hence, kin selection favours cooperation. These requirements are consistent with the empirical examples of pleiotropy in both slime moulds and bacteria. The hypotheses could also be distinguished by experimental evolution. If cooperation stabilises pleiotropy, then if selection for cooperation is removed, we would expect both pleiotropy and cooperation to be lost over time. Cooperation could be selected against by imposing low relatedness in cultures [18,54,69,70]. In contrast, if pleiotropy stabilises cooperation, then even under conditions of low relatedness, cooperation will be maintained. In addition, as we have discussed above, the hypothesis that cooperation favours pleiotropy does not necessarily require that the private trait is essential, only that it confers a sufficiently large fitness advantage.

### Conclusions

More generally, our results illustrate how selection on social traits, such as cooperation, can also shape the genetic architecture [6]. The genetic architecture is not fixed—like any aspect of an organism, it is subject to natural selection and can evolve [21,42,43,71,72]. We have shown that, especially in microbes, if cooperation is favoured, this can select for other traits to be pleiotropically linked with this cooperation. Furthermore, this is only one way in which social interactions could drive genome evolution. Other possibilities include mutualistic cooperation leading to genome degradation or gene transfers between species and selection for cheating leading to genome reduction, genome compartmentalisation, or the sequestering of cooperative traits onto mobile genetic elements [42,43,73–78].

## Methods

### Analytical model

We model an infinitely large population of haploid individuals. The population is subdivided into a large number of patches, each of size *N*. We assume that individuals interact socially within patches and that generations are nonoverlapping. At this point, we make no assumptions as to how social groups are formed except that individuals within a given patch can potentially be more related to each other than individuals chosen at random from the population. Individuals can produce, at personal cost *c*, a public good molecule that provides a benefit *b*/*N* to each individual on the patch (including the focal). Individuals can also produce a private good that is essential for reproduction: private-good nonproducers have their baseline fecundity reduced by an amount *d*. We later consider the case in which not producing the private trait leads to the individual’s death. We also assume that individuals produce a very large number of offspring, which all disperse from their natal patch to some new random patch, such that competition is global (no kin competition). Individuals carry two traits, denoted by *p* ∈ {0, 1} and *h* ∈ {0, 1}, coding for the production of the private and public good molecules, respectively. Assuming everyone’s fecundity is always strictly positive, and since competition is global, the relative fitness a focal individual *i* on patch *j* is $w_{i,j}=F_{i,j}/\overset{-}{F}$, where $\overset{-}{F}$ is the average fecundity in the population.

### Public goods cooperation

We first determine when cooperation is favoured in a scenario in which only cooperators and defectors compete with each other. We assume here that both types express the private-good trait. Hence, the expected fecundity of a focal defector *F*_*D*_ and that of a focal cooperator *F*_*C*_ are given by

$$F_{D}=1+x_{D}(N-1)\frac{b}{N}$$ (1)

and

$$F_{C}=1-c+\frac{b}{N}+x_{C}(N-1)\frac{b}{N},$$ (2)

respectively, where *x*_*D*_ and *x*_*C*_ are the expected frequencies of cooperators among the coplayers of a focal defector and cooperator, respectively. Therefore, both types might potentially have a different social environment, i.e., if *x*_*D*_ ≠ *x*_*C*_. If groups are formed randomly, then *x*_*D*_ = *x*_*C*_. Here, *x*_*C*_ − *x*_*D*_ is relatedness *r* [38] and is identical to the interpretation of relatedness as a regression coefficient of a partner genotype on the focal player’s genotype [36,37]. Because fecundity is linear in the number of cooperators (the game is of degree 1; [39]), relatedness *r* is the only necessary genetic association for describing evolutionary change in this game [38,39]. Thus, cooperators will be favoured whenever their relative fitness *w*_C_ is greater than that of defectors *w*_D_, which is if

$$\underset{r}{\underset{︸}{\left(x_{C}-x_{D}\right)}}\underset{\tilde{b}}{\underset{︸}{(N-1)\frac{b}{N}}}+\underset{-\tilde{c}}{\underset{︸}{\left(\frac{b}{N}-c\right)}}>0.$$ (3)

### Pleiotropic cooperators against private-good nonproducers

Here, we consider the case in which cooperation is linked pleiotropically to a private trait that is essential for survival and reproduction. Hence, we assume that such private-good nonproducers suffer a fecundity cost *d*. We let pleiotropic cooperators compete with such private-good nonproducers. Therefore, the fecundity of private-good nonproducers is that of defectors (i.e., Eq 1) reduced by an amount *d*, and the fecundity of pleiotropic cooperators remains similar as that of cooperators (Eq 2). Consequently, pleiotropic cooperators will be favoured over private-good nonproducers whenever

$$\underset{r}{\underset{︸}{\left(x_{C}-x_{D}\right)}}\underset{\tilde{b}}{\underset{︸}{(N-1)\frac{b}{N}}}+\underset{-\tilde{c}}{\underset{︸}{\left(\frac{b}{N}-c+d\right)}}>0.$$ (4)

### Private goods essential for reproduction

We consider now the scenario in which not producing private goods leads to the individual’s death. Private-good nonproducers can no longer access public goods, and their share is distributed equally among the remaining pleiotropic cooperators. With these assumptions, the fecundity of private-good nonproducers becomes *F*_*PN*_ = 0, and that of pleiotropic cooperators, *F*_*PC*_, becomes

$$F_{PC}=1-c+\frac{b}{N}+(N-1)\frac{b}{N}$$

=1-c+b (5)

As we can see, the fitness of pleiotropic cooperators no longer depends on what others do. Everyone receives the full benefit *b*. Individuals will be neither more nor less likely than by chance to receive benefits from kin. Thus, the indirect fitness effect in Hamilton’s rule becomes *b̃* = 0. Hence, pleiotropic cooperators will be favoured over private-good nonproducers whenever –*c̃* > 0, where –*c̃* = 1 − *c* + *b*. This condition boils down to whether expressing both the private and public traits results in a net gain or loss. However, for a population of pleiotropic cooperators to not go extinct, the average fecundity in the population should not be smaller than 1, which is whenever *b* ≥ *c*.

### Individual-based simulations

We ran individual-based simulations to determine the validity of our analytical findings under a modified, more realistic life cycle in which growth can occur locally on a patch for a certain number of generations before a dispersal event occurs. Specifically, we consider a variant of the haystack model [79]. We model a finite population of haploid individuals subdivided into *n*_p_ = 100 patches. The life cycle is as follows: (1) Each patch is colonized by *N*_*F*_ = 100 randomly selected founding individuals. Each founder produces randomly a very large number of juveniles (without mutation), which compete for space on the patch, leaving exactly *N* = 10 individuals on each patch (the others die). To vary relatedness on a continuous scale, we assume that one randomly selected individual among the *N*_*F*_ founders will leave relatively more juveniles on the patch than the others (see below). (2) Individuals interact socially within patches. Social interactions influence their fecundity/growth rate (see above). (3) Unrestricted growth occurs within patches. All offspring survive, and parents die (nonoverlapping generations). (4) Steps 2 and 3 are repeated over *k* = 10 generations. This growth phase was implemented to simulate periods of local growth between migration events, typical of many microbial species. (v) Individuals disperse globally (every *k* generations), and exactly *N*_*F*_ founders are randomly selected from the completely mixed global pool to colonise each patch. The remaining individuals die. Unless stated otherwise, we start our simulations with all genotypes in equal proportion. Each parameter combination is run over 10^5^ generations and replicated 16 times.

#### Patch colonisation and genetic assortment

In our simulations, we varied relatedness *r* across the entire range between 0 and 1. To do so, we chose a modified colonisation pattern than in the standard haystack model, because in the latter, patches are founded by either 1, 2, 3, …, or *N*_*F*_ founders, which only generates relatedness values of *r* = 1, 1/2, 1/3, …, 1/*N*_*F*_, respectively.

The colonisation procedure is as follows. Every *k* generations, all individuals disperse globally to form a completely mixed pool of potential founders. For each patch, *N*_*F*_ = 100 individuals are randomly selected from the global pool and compete for reproduction on the patch. We assume that one random founder among the *N*_*F*_ founders arrives first on the patch and reproduces until the *N*_*F*_ − 1 secondary founders arrive and reproduce as well. Founders reproduce until there are *N* = 10 offspring on each patch. In order to vary relatedness between patch members, we vary the time left before the *N*_*F*_ − 1 secondary founders arrive on the patch. This influences the proportion of the *N* initial offspring that will descend from the first founder, and hence, it influences the probability that two randomly chosen offspring from the same patch descend from the same parent, namely relatedness *r*.

To implement this, we assign fitness *v* to the first founder and (1 − *v*)/(*N*_*F*_ − 1) to each of the remaining *N*_*F*_ − 1 secondary founders. Then, exactly *N* founders are sampled with replacement to be the parent of an offspring, in proportion to their assigned fitness. For example, if *v* = 1/*N*_*F*_, the first founder is as likely to reproduce as every other secondary founder, and the probability that two randomly chosen offspring have the same parent—i.e., relatedness *r* (*r* at generation *k* = 1)—will be 1/*N*_*F*_. More generally, the relationship between *v* and *r* is given by *r* = *v*^2^ + (1 − *v*)^2^/ (*N*_*F*_ − 1), where *v*^2^ and (1 − *v*)^2^/ (*N*_*F*_ − 1) are the probabilities that two randomly chosen offspring in a given patch both descend from the first founder and the same secondary founder, respectively.

To determine the values of *v* that correspond to our desired values of *r* (i.e., 0.01, 0.05, 0.1, …, 1) we solved *r* = *v*^2^ + (1 − *v*)^2^/(*N*_*F*_ − 1) for *v* and then plugged in our values of *r*. To confirm that our colonisation procedure yielded the expected values of *r*, we ran neutral simulations with only two genotypes—cheats and cooperators—and measured *r* as the difference in frequency of cooperators among the *N* − 1 partners between cooperators and cheats at the end of step 1 of the life cycle. S2 Fig confirms that our values of *v* yielded the predicted values of *r* in this neutral model. This figure also shows that relatedness increases during the growth phase (i.e., during the *k* = 10 generations of unrestricted growth), especially when the initial *r* is low. At high initial *r*, however, relatedness during the growth phase is mostly eroded by mutations (S2 Fig).

#### Growth

After the social interactions, unrestricted growth occurs on each patch (i.e., there is neither competition for space nor regulation; thus, patch size can potentially grow to infinity). We assume that not producing the private good leads to the individual’s death. Each individual that expresses the private trait produces one offspring plus a random number of clonal offspring (with mutations, see below), which is Poisson distributed around the payoff acquired during social interactions incremented by a baseline growth rate *g*. Parent individuals die. During reproduction (step 3 of the life cycle), each offspring produced undergoes a mutation with probability *μ* = 0.001. In this case, the mutation occurs randomly at one of the two loci.

## Supporting information

S1 Text Supplementary information. In this file, we first explore how sensitive our result that pleiotropy does not stabilise cooperation is to our model assumptions. Specifically, we explore different mutational pathways for pleiotropy. Second, using individual-based simulations, we explicitly model the pleiotropic link between cooperation and the private trait by assuming that this link is controlled by a third independent locus.
(PDF)

S1 Table The importance of pleiotropy in stabilising cooperation in the literature. In this table, we list previous claims about the general importance of pleiotropy in stabilising cooperation.
(PDF)

S2 Table The genotypes used in our explicit model of pleiotropy. In this table, we list all the possible genotypes we used in our explicit model in which the pleiotropic link takes the form of a universal regulator.
(PDF)

S1 Fig Pleiotropy can help stabilise all forms of social trait. We consider all possible forms of social interaction by considering a trait that has a fecundity effect −*c* for the individual performing it and a fecundity effect *b* that is shared amongst all the members of the group. If *b* > 0, then the trait is helpful, providing a benefit to both self and others (public good), whereas if *b* < 0, then the trait is harming and costly to both self and others. If *c* > 0, then the trait has some fecundity cost to perform, but if *c* < 0, then performing the trait provides some fecundity benefit. The area to the left of the dashed line shows where the trait will be favoured when the trait also has some pleiotropic private benefit. The dark coloured area is when the trait would be favoured without the pleiotropic private trait. The light coloured area represents the extent to which pleiotropy can help stabilise social traits. We denote the lifetime fitness cost and benefits by *c̃* = *c* − *b*/*N* − *d* and *b̃* = (*N* − 1) *b*/*N*, respectively. We follow Rousset [80] by dividing the figure with the lines *c̃* = 0 (*c* = *b*/*N* + *d*) and *b̃* = 0 (*b* = 0) into the four classes of social behaviours—mutually beneficial, altruism, spite, and selfishness. This classification holds for when the trait has some pleiotropic private benefit. Parameters: *N* = 10, *r* = 0.5, *d* = 0.4.
(TIF)

S2 Fig Relatedness in neutral runs. Shown is the average relatedness of 16 replicates during the last 20,000 generations, as a function of the fitness *v* of the first founder. Relatedness increases between step 1 and step *k* = 10 of the life cycle, especially at low initial relatedness. At higher values of initial *r*, relatedness is mostly eroded by mutations. This increase in relatedness during the growth phase is explained as follows. Consider, for example, a neutral model in which the number of cooperators and cheats doubles in each patch. Hence, the within-patch proportion of cooperators remains unchanged. However, the frequency of cooperators among the *N* − 1 partners experienced by a focal cooperator increases, and that of a focal cheat decreases. For example, if at generation *t*, a patch contains 2 cooperators and 1 cheat. Then, a focal cooperator has 1 cooperator and 1 cheat in its group (0.5:0.5), whereas a focal cheat has 2 cooperators and 0 cheats in its group (1:0). At generation *t* + 1, population size doubles. Hence, a focal cooperator now has 3 cooperators and 2 cheats in its group (0.6:0.4), whereas a focal cheat now has 4 cooperators and 1 cheat in its group (0.8:0.2). The frequency of cooperators among the social partners of a focal cooperator increases, and that of a focal cheat decreases. Therefore, relatedness increases as population size increases, as long as there remains some nonhomogeneous groups (in homogeneous groups, the frequency of cooperators among social partners no longer changes). Parameters: *c* = 0.1, *g* = 0.5, *μ* = 0.001.
(TIF)

S3 Fig Pleiotropy promotes cooperation and anything else. Pleiotropic cooperators compete with noncooperative private nonproducers. Shown is the average pleiotropy as a function of the production cost *c* and the public good benefit *b*. Pleiotropy prevails even when producing public goods is harmful to both the actor and its partners—i.e., *b* ≤ 0—as long as expressing both the private and social traits leads to a fitness that is greater or equal to 1. Otherwise, the population goes extinct (white area). Parameters: *c* = 0.1, *g* = 0.5, *μ* = 0.001.
(TIF)

S4 Fig Pleiotropy and the cost of not producing the private good. Pleiotropic cooperators compete with noncooperative private nonproducers. Pleiotropy prevails as long as expressing both the private and cooperation trait leads to a sufficiently better growth rate. Otherwise, noncooperative private nonproducers prevail (white area). In these runs, relatedness, *r* = 0.01, which represents the most difficult condition for nonpleiotropic cooperation to evolve in our simulation. The baseline growth rate of pleiotropic cooperators *g* = 0.5. Parameters: *c* = 0.1, *μ* = 0.001.
(TIF)

S5 Fig Pleiotropy and a mutational bias of 10. Losing a function (i.e., cooperation, private trait, and pleiotropy) is 10 times more likely than gaining one. For example, if a mutation occurs in a nonpleiotropic cooperator, it has 10 times more chances to lead to a loss of either cooperation or private production than a gain of the pleiotropic link. As before, cooperation only evolves when Hamilton’s rule is satisfied. However, pleiotropy is only favoured under high relatedness and large cooperation benefits and a high mutation rate. Panels (a) and (c) show the frequency of cooperation, and panels (b) and (d) show their respective proportion of pleiotropic cooperators. The dashed line represents the analytical prediction for when Hamilton’s rule is satisfied, assuming that migration occurs every generation (i.e., *k* = 1 in Eq 3 in the main text). In all panels, all genotypes and mutations I and II in Fig 3 of the main text are allowed. Parameters: *c* = 0.1, *g* = 0.5, *k* = 10.
(TIF)

S6 Fig Pleiotropy and a mutational bias of 100. Losing a function (i.e., cooperation, private trait, and pleiotropy) is 100 times more likely than gaining one. For example, if a mutation occurs in a nonpleiotropic cooperator, it has 100 times more chances to lead to a loss of either cooperation or private production than a gain of the pleiotropic link. As before, cooperation only evolves when Hamilton’s rule is satisfied. However, pleiotropy is only favoured with relatedness and cooperation benefits close to 1 and a high mutation rate. Panels (a) and (c) show the frequency of cooperation, and panels (b) and (d) show their respective proportion of pleiotropic cooperators. The dashed line represents the analytical prediction for when Hamilton’s rule is satisfied, assuming that migration occurs every generation (i.e., *k* = 1 in Eq 3 in the main text). In all panels, all genotypes and mutations I and II in Fig 3 in the main text are allowed. Parameters: *c* = 0.1, *g* = 0.5, *k* = 10.
(TIF)

S7 Fig Cooperation and pleiotropy frequencies in different scenarios. Each column represents a distinct scenario, whose corresponding genetic architecture is shown at the top. In (a-c) and (j-l), pleiotropy can revert to nonpleiotropic cooperation (mutation II in Fig 3 in the main text). In (d-f) and (j-l), mutations on the cooperation trait in pleiotropic individuals are possible and generate cheats. In (j-l), mutations on the private trait in pleiotropic individuals are possible. Pleiotropy prevails only when Hamilton’s rule is satisfied when in competition with all nonpleiotropic genotypes (Fig 3 in the main text). Whenever pleiotropy cannot revert to a two-regulator system (panels d-i), the population is entirely invaded by pleiotropic cooperators. Parameters: *c* = 0.1, *g* = 0.5.
(TIF)

S8 Fig Genetic elements in the explicit model. We model more explicitly the pleiotropic link between the private and cooperation genes. Each gene is expressed only if both the expressing version of the allele (*C* or *P*) and the corresponding expressing version of its regulator (filled *regA* for the private trait and filled *regB* for the cooperation trait) are present. We assume that each regulator can be lost and that the private regulator, *regA*, can become pleiotropic by being able to regulate both genes at the same time (pointy orange circle). During reproduction, each regulator mutates independently with probability *μ*_R_, and each gene mutates with probability *μ*. We assume that the private trait is essential, so in case the private regulator and/or private gene is lost through mutation, the individual dies. All the possible genotypes resulting from these elements and their corresponding phenotypes are listed in S2 Table.
(TIF)

S9 Fig Cooperation and pleiotropy when the pleiotropic link is explicitly modelled as a universal regulator. Panels (a) and (c) show the frequency of cooperation, and panels (b) and (d) show their respective relative proportion of pleiotropic cooperators (i.e., frequency of genotype number g20 + g24 over the sum of frequencies of the genotype number g16, g20, g24). The dashed line represents the analytical prediction *r*(*N* − 1)*b*/*N* = *c* − *b*/*N*, for when Hamilton’s rule is satisfied, assuming that migration occurs every generation (i.e., *k* = 1). Parameters: *n*_*p*_ = 500, *c* = 0.1, *g* = 0.5, *k* = 10, *μ*_R_ = 0.001.
(TIF)

S10 Fig Proportion of cheats produced by mutation when the pleiotropic link is explicitly modelled as a universal regulator. All panels show the proportion of cheats produced during a single growth phase for different values of the cooperation benefit *b* and mutation rate *μ*. Each patch is started with 10 cooperators, and the *x* axis shows the number of those cooperators for which cooperation was pleiotropically linked to an essential private trait, using pleiotropic cooperators with genotype number g20 (a-b) or g24 (c-d). Increased pleiotropy leads to a decreased accumulation of cheats. Each bar represents the average of 10^4^ patches. Parameters: *c* = 0.1, *g* = 0.5, *k* = 10, *μ*_R_ = 0.001.
(TIF)

S11 Fig Cooperation and pleiotropy in neutral runs when the pleiotropic link is explicitly modelled as a universal regulator. Panels (a) and (c) show the frequency of cooperation, which ranges between 0.36 and 0.38. This is because there are three cooperative genotypes among a total of 8 viable genotypes (S2 Table), with 3/8 = 0.375. Panels (b) and (d) show their respective relative proportion of pleiotropic cooperators (i.e., frequency of genotype number g20 + g24 over the sum of frequencies of the genotype number g16, g20, g24). The dashed line represents the analytical prediction *r*(*N* − 1)*b*/*N* = *c* − *b*/*N*, for when Hamilton’s rule is satisfied, assuming that migration occurs every generation (i.e., *k* = 1). All runs are neutral with respect to the cooperation trait but not the essential private trait; i.e., all individuals that express the essential private trait have fitness 1 + *g*, whereas the others die. Parameters: *n*_*p*_ = 500, *g* = 0.5, *k* = 10, *μ*_R_ = 0.001.
(TIF)

S12 Fig Pleiotropy between two privately beneficial traits. As in our baseline scenario, the private trait is essential. However, the cooperation trait is replaced by a second privately beneficial trait (with *b* = 0 and *c* < 0). As a result, individuals expressing both private traits can either be pleiotropic or nonpleiotropic. In both panels, individuals with a pleiotropic link between two private traits are never more common than individuals without this pleiotropic link, as their proportion never exceeds 50%. Parameters: *b* = 0, *g* = 0.5, *k* = 10.
(TIF)

S13 Fig Cooperation and pleiotropy with fluctuating benefit *b*. The benefit of cooperation is alternating between 0 and the value shown on the *y* axis every 10 generations (i.e., every growth phase). Cooperation is less likely to evolve under such fluctuating environment, and pleiotropy only evolves when Hamilton’s rule is satisfied. Panels (a) and (c) show the frequency of cooperation, and panels (b) and (d) show their respective proportion of pleiotropic cooperators. The dashed line represents the analytical prediction for when Hamilton’s rule is satisfied, assuming that the benefit is *b*/2 and that migration occurs every generation (i.e., *k* = 1; and substituting *b* = *b*/2 in Eq 3 in the main text). In all panels, all genotypes and mutations I and II in Fig 3 of the main text are allowed. Parameters: *c* = 0.1, *g* = 0.5, *k* = 10.
(TIF)

S14 Fig Cooperation with and without pleiotropy and fluctuating benefit *b*. The benefit of cooperation is alternating between 0 and the value shown on the *y* axis every 10 generations (i.e., every growth phase). Cooperation is more likely to evolve if pleiotropy is allowed. In (a) and (b), pleiotropic cooperators are allowed (all genotypes and mutations I and II in Fig 3 in the main text). In (c) and (d), pleiotropic cooperators are replaced by nonpleiotropic cooperators (this maintains a similar ratio of cooperative strategies to when pleiotropic cooperators are present). Panels (e) and (f) show the difference in cooperation frequency between (a) and (c) and (b) and (d), respectively. The dashed line represents the analytical prediction for when Hamilton’s rule is satisfied, assuming that the benefit is *b*/2 and that migration occurs every generation (i.e., *k* = 1; and substituting *b* = *b*/2 in Eq 3 in the main text). Parameters: *c* = 0.1, *g* = 0.5, *k* = 10.
(TIF)

S15 Fig Cooperation and pleiotropy with fluctuating population structure. Population structure (relatedness *r*) is alternating between 0.01 and the value shown on the *x* axis every 10 generations (i.e., every growth phase). Cooperation is less likely to evolve under such fluctuating environment, and pleiotropy only evolves when Hamilton’s rule is satisfied. Panels (a) and (c) show the frequency of cooperation, and panels (b) and (d) show their respective proportion of pleiotropic cooperators. The dashed line represents the analytical prediction for when Hamilton’s rule is satisfied assuming that relatedness is (*r* + 0.01)/2 and that migration occurs every generation (i.e., *k* = 1; and substituting *r* = [*r* + 0.01)/2 in Eq 3 in the main text). In all panels, all genotypes and mutations I and II in Fig 3 of the main text are allowed. Parameters: *c* = 0.1, *g* = 0.5, *k* = 10.
(TIF)

S16 Fig Cooperation with and without pleiotropy and fluctuating population structure. Population structure (relatedness *r*) is alternating between 0.01 and the value shown on the *x* axis every 10 generations (i.e., every growth phase). Cooperation is more likely to evolve if pleiotropy is allowed. In (a) and (b), pleiotropic cooperators are allowed (all genotypes and mutations I and II in Fig 3 of the main text). In (c) and (d), pleiotropic cooperators are replaced by nonpleiotropic cooperators (this maintains a similar ratio of cooperative strategies to when pleiotropic cooperators are present). Panels (e) and (f) show the difference in cooperation frequency between (a) and (c) and (b) and (d), respectively. The dashed line represents the analytical prediction for when Hamilton’s rule is satisfied assuming that relatedness is (*r* + 0.01)/2 and that migration occurs every generation (i.e., *k* = 1; and substituting *r* = [*r* + 0.01]/2 in Eq 3 in the main text). Parameters: *c* = 0.1, *g* = 0.5, *k* = 10.
(TIF)

S17 Fig Cooperation promotes pleiotropy under relaxed assumptions. Panels (a) and (c) show the frequency of cooperation for different mutation rates *μ*, and panels (b) and (d) show their respective proportion of pleiotropy relative to all cooperative genotypes (i.e., pleiotropic cooperators, nonpleiotropic cooperators, and cooperative private nonproducers). The dashed lines represent the analytical prediction for when Hamilton’s rule is satisfied assuming that migration occurs every generation (i.e., *k* = 1; Eq 3 in the main text). Panel (e) shows the proportion of cheats produced by mutation and growth during a single growth phase (mutation rate *μ* = 0.005) for different values of the cooperation benefit *b*. Each patch is started with 10 cooperators, and the *x* axis shows the number of those cooperators for which cooperation was pleiotropically linked to an essential private trait. Increased pleiotropy leads to a decreased accumulation of cheats, but to a lesser extent than when mutations in pleiotropic individuals cannot generate cheats (compare panel [c] with Fig 4C in the main text). Each bar represents the average of 10^4^ patches. Parameters: *c* = 0.1, *g* = 0.5, *k* = 10.
(TIF)
