# Supplementary material for: Pleiotropy, cooperation, and the social evolution of genetic architecture
Source: PLoS Biol. 2018 Oct 25;16(10):e2006671. doi: 10.1371/journal.pbio.2006671 (PMC6219813; doi:10.1371/journal.pbio.2006671)
Supplement: S1 Text — In this file, we first explore how sensitive our result that pleiotropy does not stabilise cooperation is to our model assumptions. Specifically, we explore different mutational pathways for pleiotropy. Second, using individual-based simulations, we explicitly model the pleiotropic link between cooperation and the private trait by assuming that this link is controlled by a third independent locus. (PDF) [file pbio.2006671.s001.pdf]

S1 Text. Supplementary Information

Pleiotropy, cooperation and the social evolution of genetic architecture

Miguel dos Santos[1*], Melanie Ghoul[1] & Stuart A. West[1]

[1]Department of Zoology, University of Oxford, South Parks Road, Oxford OX1 3PS, UK.

This files includes

- Supplementary analyses
- Supplementary references

1. Supplementary analyses

In this section, we first explore how sensitive our result that pleiotropy does not stabilise cooperation is to our model assumptions. Specifically, we explore different mutational pathways for pleiotropy. Second, using individual-based simulations, we explicitly model the pleiotropic link between cooperation and the private trait, by assuming that this link is controlled by a third independent locus.

1.1 Different mutational pathways

In the main text, we showed that pleiotropy does not stabilise cooperation unless the genetic architecture cannot evolve: the link between cooperation and an essential private trait cannot be broken. Here, we test how robust this result is with respect to changes in the mutational pathways, or mutational accessibility, allowed in our simulations. In the model presented in the main text, pleiotropy could be gained or lost due to a mutation in individuals who bear both the private and cooperation traits, and only one mutation in pleiotropic cooperators could lead to the individual expressing neither traits (Fig 3). Here, we explore 3 different scenarios:

In scenario (i), the only difference with our baseline model (main text) is that pleiotropy cannot be lost (no mutational accessibility), so the only mutation in pleiotropic cooperators, which occurs with probability μ, leads to the loss of the pleiotropic regulator, and hence, the individual's death, because the private trait is no longer expressed. This is an extreme scenario because pleiotropy becomes an attractor, as no other genotypes can be generated from pleiotropic cooperators. Therefore, pleiotropy entirely invades the population, but only when Hamilton's rule is satisfied (S7g-l Fig).

In scenario (ii), we allow mutations on both the cooperation and private trait in pleiotropic cooperators in addition to mutations on the pleiotropic regulator, so that with probability μ, a mutation occurs and leads (randomly) to either (a) cheats, (b) cooperative private non-producers, (c) non-pleiotropic cooperators, or (d) uncooperative private non-producers. This case can be viewed as the least constrained scenario, because all mutations are allowed, and pleiotropy can be lost. As before, pleiotropy does not stabilise cooperation in this case (S7j-l Fig). Instead, cooperation promotes pleiotropy, as shown by greater proportions of pleiotropic cooperators relative to other cooperative genotypes when cooperation is strongly favoured, especially with a high mutation rate (S17b and d Fig). We also found that more pleiotropic cooperators within patches result in lower proportions of cheaters at the end of the growth phase, confirming our cheat-load hypothesis (S17e Fig).

In scenario (iii), we prevent pleiotropy from being lost (mutation II in Fig. 3 of the main text), but we also allow mutations on the cooperation trait, so that cheats can be generated. Hence, in pleiotropic cooperators, a mutation occurs with probability μ, and leads (randomly) to either (a) uncooperative private non-producers (death), or (b) cheats. When Hamilton's rule is satisfied, this mutational pathway results in pleiotropy being an attractor as in scenario (i). This is because pleiotropic cooperators generate either unviable individuals or cheats who are favoured locally, but counter-selected globally. As a consequence, when Hamilton's rule is satisfied, the population is mostly composed of pleiotropic cooperators (S7d-f Fig).

1.2 An explicit model of pleiotropy

In our baseline model presented in the main text, we have not explicitly modelled how the pleiotropic link could be formed between the cooperation and private traits. However, the details of how this link could form might influence our conclusion on how pleiotropy stabilises cooperation,

or whether cooperation promotes pleiotropy. Here, we test whether these results are robust to changes in the way the pleiotropy link is formed.

We explicitly model the pleiotropic link between cooperation and a private trait. We introduce two regulators, *regA* and *regB*, which control the expression of the private and cooperation traits, respectively, when these traits are present (i.e. allele *P* and *C*, respectively; S8 Fig). As a result, the expression of a trait requires both the expressing allele and its regulator to be present. Each regulator can either be present or not present (0/1). In addition, we assume that the private regulator *regA* can become a universal regulator, i.e. with pleiotropic effects. That is, the regulator can activate both the private and cooperation traits, at the same time. During reproduction, each regulator undergoes mutations independently with probability μ_R = 0.001. With these assumptions, the total number of genotypes is 3×2×2×2 = 24. This scenario reflects a situation where both regulators are independent from each other. Hence, we chose to let them be affected by mutations independently, such that a mutation on *regA* does not influence *regB*. So, *regB* can remain active (albeit redundant) even if *regA* becomes pleiotropic. We assume that only the private regulator *regA* can become universal for simplicity, otherwise the number of genotypes becomes considerably larger.

All 24 genotypes are presented in S2 Table. As in our baseline model, we assume that the private trait is essential, so that not expressing it leads to the individual's death. As a result, only 8 genotypes are viable, 5 uncooperative and 3 cooperative ones. Among the 3 cooperative genotypes, 2 are pleiotropic. Hence, we take a proportion of pleiotropic cooperators above 2/3 as indication that pleiotropy has an advantage over non-pleiotropic cooperators. Neutral simulations show that the proportion of pleiotropic cooperators relative to all 3 cooperative genotypes never exceeded 0.7 (S11 Fig), confirming that mutations do not generate more pleiotropic cooperators than expected.

S9-10 Figs show the results of this explicit model. As in our baseline model (main text), pleiotropy does not stabilise cooperation (S9a and c Fig). In addition, we also found that pleiotropic cooperators were more common relative to non-pleiotropic cooperators when cooperation is strongly favoured (top-right of S9b and S9d Fig). As before, pleiotropy is favoured in this model because it reduces the local cheat-load, as shown by lower proportions of cheats generated during a single growth phase, when the number of pleiotropic cooperators increases (S10 Fig). However, this effect is smaller than in our baseline model, as shown by small differences in the proportion of cheats with increasing number of pleiotropic cooperators (S10 Fig).

Supplementary references

Rousset F (2004) Genetic structure and selection in subdivided populations. Princeton University Press, Princeton.
