# Supplementary material for: Pleiotropy, cooperation, and the social evolution of genetic architecture
Source: PLoS Biol. 2018 Oct 25;16(10):e2006671. doi: 10.1371/journal.pbio.2006671 (PMC6219813; doi:10.1371/journal.pbio.2006671)
Supplement: S1 Table — In this table, we list previous claims about the general importance of pleiotropy in stabilising cooperation. (PDF) [file pbio.2006671.s002.pdf]

S1 Table. Previous claims about the general importance of pleiotropy in stabilising cooperation.

**Reference**	**Importance of pleiotropy for stabilising cooperation**
Foster et al., 2004 (p. 695)	Pleiotropy is placed among other mechanisms such as (high relatedness) kin selection
Dandekar et al., 2012 (p. 266)	Pleiotropy is placed alongside single cell/bottle-neck dispersal (kin selection) mechanisms
Bruger & Waters, 2015 (p. 3)	'metabolic constraint' (private traits linked to cooperation) is one of the five mechanisms to maintain cooperation
Frénoy et al., 2015 (abstract)	"Our results […] uncover an important genetic mechanism for the evolution and maintenance of cooperation."
Wang et al., 2015 (p. 2189)	Pleiotropy (co-regulation) is placed alongside kin selection and policing.
Mitri & Foster, 2016 (p. 488)	"Pleiotropy […] is thought to be a key mechanism for stabilizing cooperation against cheater mutants"
Majerczyk et al., 2016 (p. 1-2)	Different forms of pleiotropy (metabolic constraint and coregulation) are given as two of the three ways that have been shown to stabilise cooperative quorum sensing.
Özkaya et al., 2017 (p. 3)	Pleiotropy is given as one of the main ways to prevent cheating, alongside factors such as spatial structure (kin selection) and policing.
Asfahl & Shuster, 2017 (p. 99)	Pleiotropy is an 'evolutionary force' in addition to kin selection
Chisholm et al., 2018 (p. 47)	"Genetic interaction […] may play a key role in the maintenance of altruistic traits."
