# Supplementary material for: Pleiotropy, cooperation, and the social evolution of genetic architecture
Source: PLoS Biol. 2018 Oct 25;16(10):e2006671. doi: 10.1371/journal.pbio.2006671 (PMC6219813; doi:10.1371/journal.pbio.2006671)
Supplement: S2 Table — In this table, we list all the possible genotypes we used in our explicit model in which the pleiotropic link takes the form of a universal regulator. (PDF) [file pbio.2006671.s003.pdf]

S2 Table. All possible genotypes in our explicit model where the pleiotropic link takes the form of a universal regulator (i.e., regulating simultaneously the private and cooperation trait).

Regulator A (private)	Regulator B (cooperation)	Private loci	Cooperation loci	Phenotype	Genotype number
0	0	0	0	unviable	g1
0	0	0	1	unviable	g2
0	0	1	0	unviable	g3
0	0	1	1	unviable	g4
0	1	0	0	unviable	g5
0	1	0	1	unviable	g6
0	1	1	0	unviable	g7
0	1	1	1	unviable	g8
1	0	0	0	unviable	g9
1	0	0	1	unviable	g10
1	0	1	0	**Cheat**	g11
1	0	1	1	**Cheat**	g12
1	1	0	0	unviable	g13
1	1	0	1	unviable	g14
1	1	1	0	**Cheat**	g15
1	1	1	1	**Cooperator**	g16
2	0	0	0	unviable	g17
2	0	0	1	unviable	g18
2	0	1	0	**Pleiotropic cheat**	g19
2	0	1	1	**Pleiotropic cooperator**	g20
2	1	0	0	unviable	g21
2	1	0	1	unviable	g22
2	1	1	0	**Pleiotropic cheat**	g23
2	1	1	1	**Pleiotropic cooperator**	g24

0: absent; 1: present; 2: universal/pleiotropic regulator
