# Supplementary figures and images for: Pleiotropy, cooperation, and the social evolution of genetic architecture
Source: PLoS Biol. 2018 Oct 25;16(10):e2006671. doi: 10.1371/journal.pbio.2006671 (PMC6219813; doi:10.1371/journal.pbio.2006671)

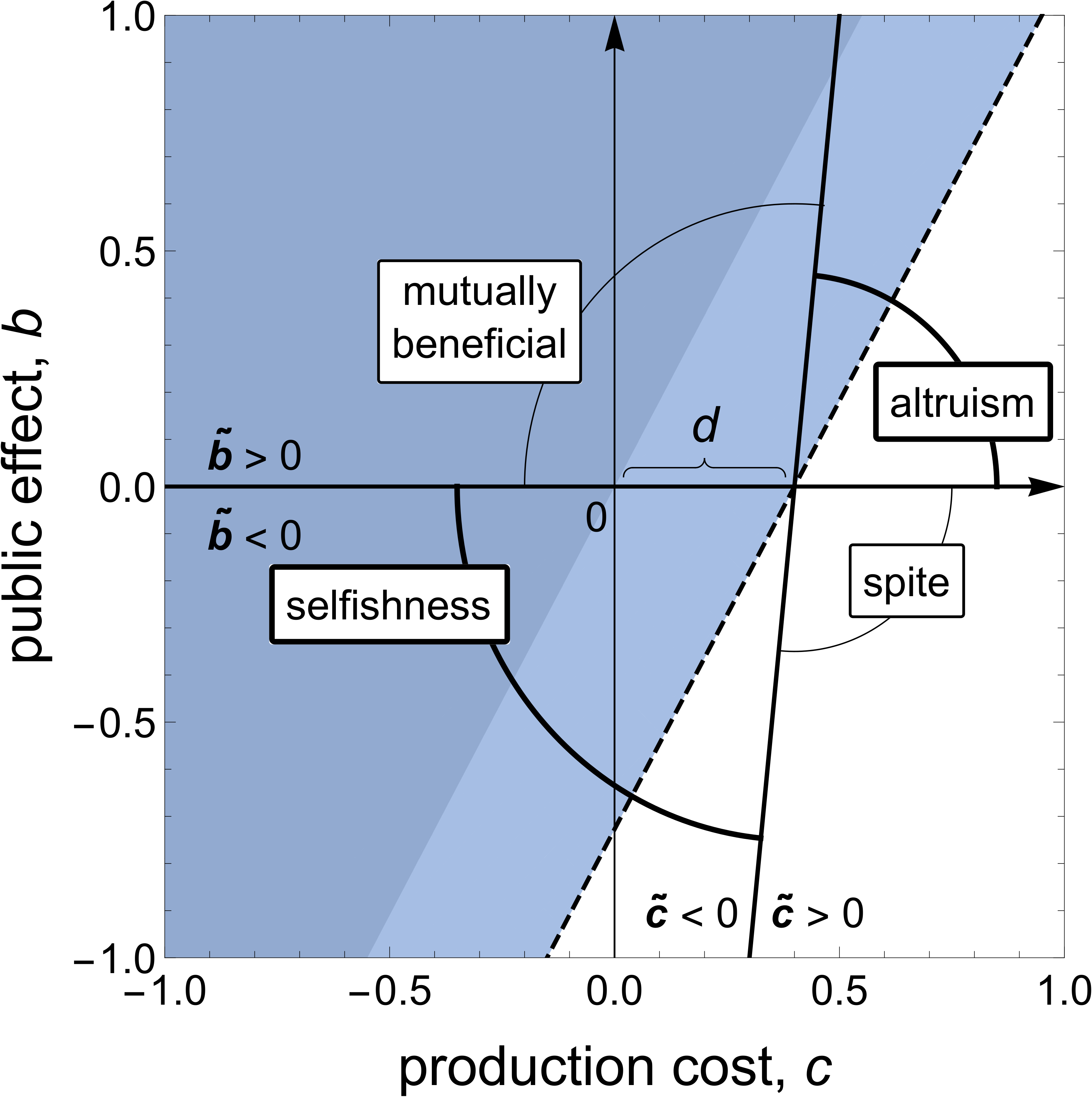

Supplement: S1 Fig — We consider all possible forms of social interaction by considering a trait that has a fecundity effect −c for the individual performing it and a fecundity effect b that is shared amongst all the members of the group. If b > 0, then the trait is helpful, providing a benefit to both self and others (public good), whereas if b < 0, then the trait is harming and costly to both self and others. If c > 0, then the trait has some fecundity cost to perform, but if c < 0, then performing the trait provides some fecundity benefit. The area to the left of the dashed line shows where the trait will be favoured when the trait also has some pleiotropic private benefit. The dark coloured area is when the trait would be favoured without the pleiotropic private trait. The light coloured area represents the extent to which pleiotropy can help stabilise social traits. We denote the lifetime fitness cost and benefits by c̃ = c − b/N − d and b̃ = (N − 1) b/N, respectively. We follow Rousset [80] by dividing the figure with the lines c̃ = 0 (c = b/N + d) and b̃ = 0 (b = 0) into the four classes of social behaviours—mutually beneficial, altruism, spite, and selfishness. This classification holds for when the trait has some pleiotropic private benefit. Parameters: N = 10, r = 0.5, d = 0.4. (TIF) [file pbio.2006671.s004.tif]

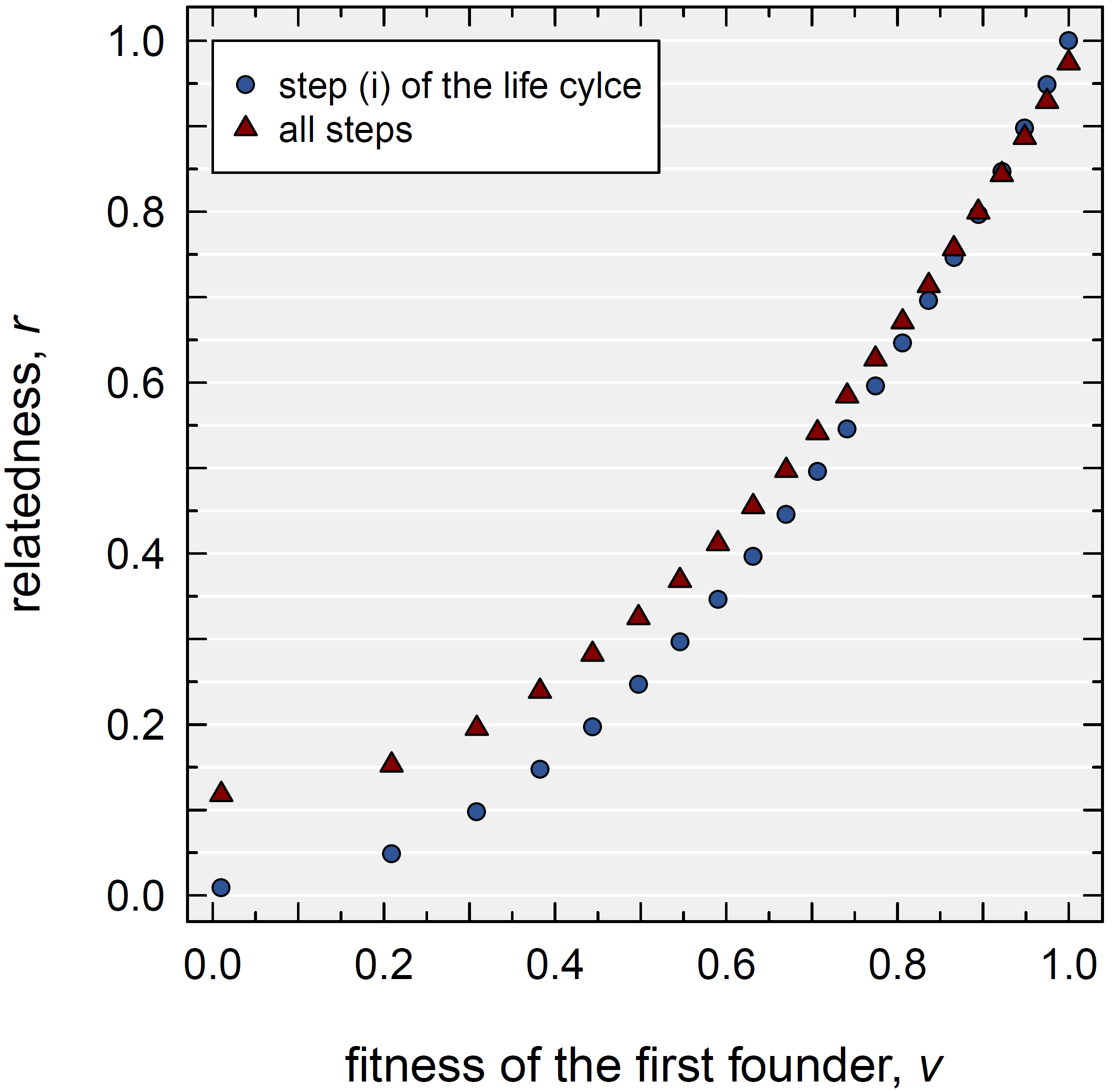

Supplement: S2 Fig — Shown is the average relatedness of 16 replicates during the last 20,000 generations, as a function of the fitness v of the first founder. Relatedness increases between step 1 and step k = 10 of the life cycle, especially at low initial relatedness. At higher values of initial r, relatedness is mostly eroded by mutations. This increase in relatedness during the growth phase is explained as follows. Consider, for example, a neutral model in which the number of cooperators and cheats doubles in each patch. Hence, the within-patch proportion of cooperators remains unchanged. However, the frequency of cooperators among the N − 1 partners experienced by a focal cooperator increases, and that of a focal cheat decreases. For example, if at generation t, a patch contains 2 cooperators and 1 cheat. Then, a focal cooperator has 1 cooperator and 1 cheat in its group (0.5:0.5), whereas a focal cheat has 2 cooperators and 0 cheats in its group (1:0). At generation t + 1, population size doubles. Hence, a focal cooperator now has 3 cooperators and 2 cheats in its group (0.6:0.4), whereas a focal cheat now has 4 cooperators and 1 cheat in its group (0.8:0.2). The frequency of cooperators among the social partners of a focal cooperator increases, and that of a focal cheat decreases. Therefore, relatedness increases as population size increases, as long as there remains some nonhomogeneous groups (in homogeneous groups, the frequency of cooperators among social partners no longer changes). Parameters: c = 0.1, g = 0.5, μ = 0.001. (TIF) [file pbio.2006671.s005.tif]

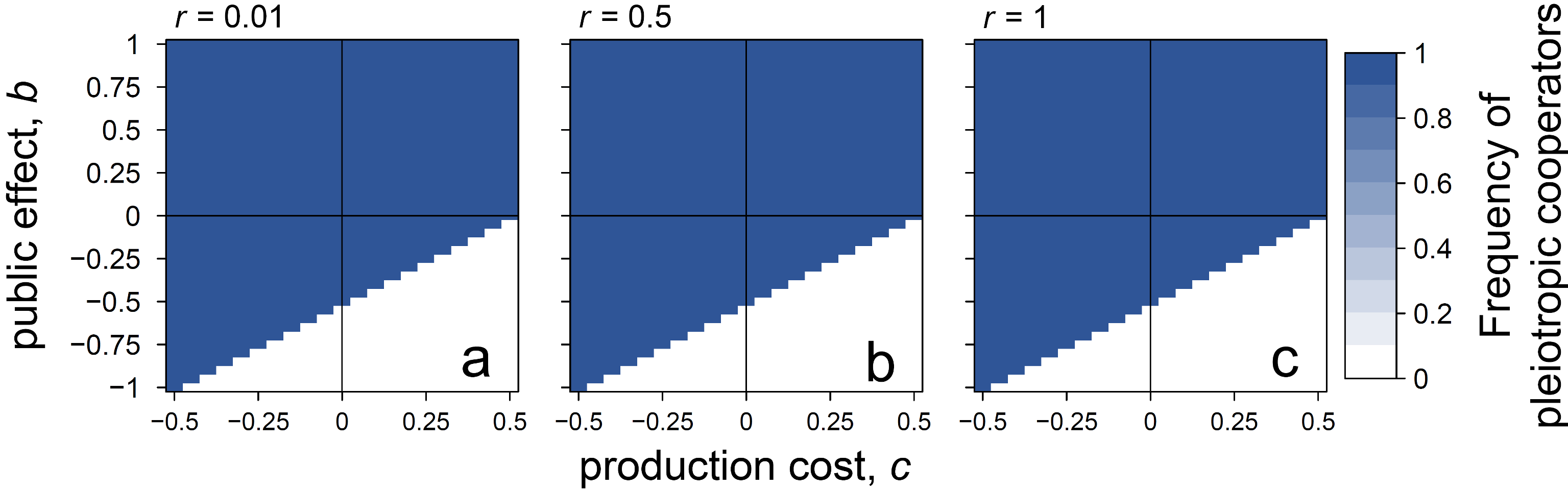

Supplement: S3 Fig — Pleiotropic cooperators compete with noncooperative private nonproducers. Shown is the average pleiotropy as a function of the production cost c and the public good benefit b. Pleiotropy prevails even when producing public goods is harmful to both the actor and its partners—i.e., b ≤ 0—as long as expressing both the private and social traits leads to a fitness that is greater or equal to 1. Otherwise, the population goes extinct (white area). Parameters: c = 0.1, g = 0.5, μ = 0.001. (TIF) [file pbio.2006671.s006.tif]

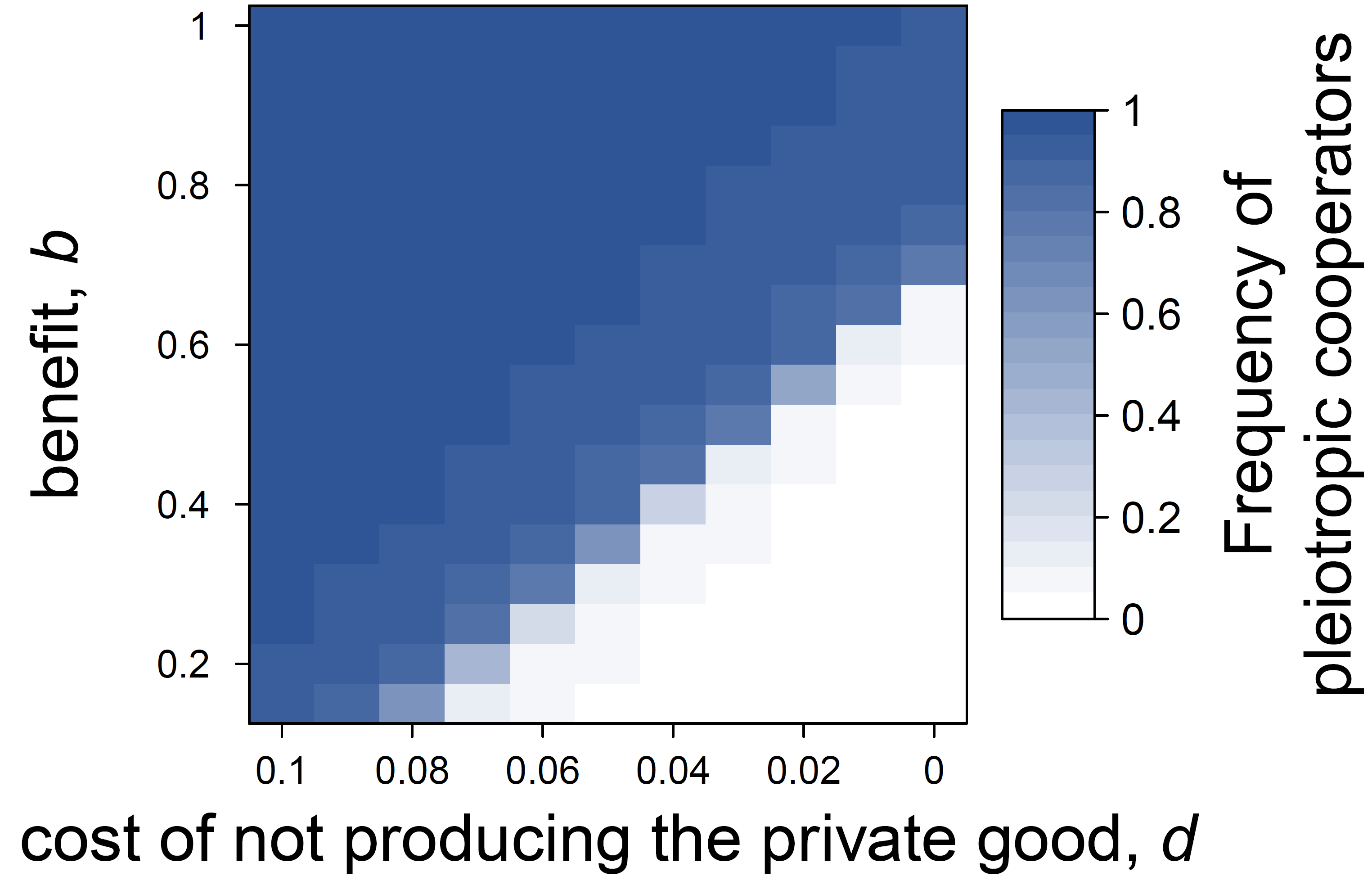

Supplement: S4 Fig — Pleiotropic cooperators compete with noncooperative private nonproducers. Pleiotropy prevails as long as expressing both the private and cooperation trait leads to a sufficiently better growth rate. Otherwise, noncooperative private nonproducers prevail (white area). In these runs, relatedness, r = 0.01, which represents the most difficult condition for nonpleiotropic cooperation to evolve in our simulation. The baseline growth rate of pleiotropic cooperators g = 0.5. Parameters: c = 0.1, μ = 0.001. (TIF) [file pbio.2006671.s007.tif]

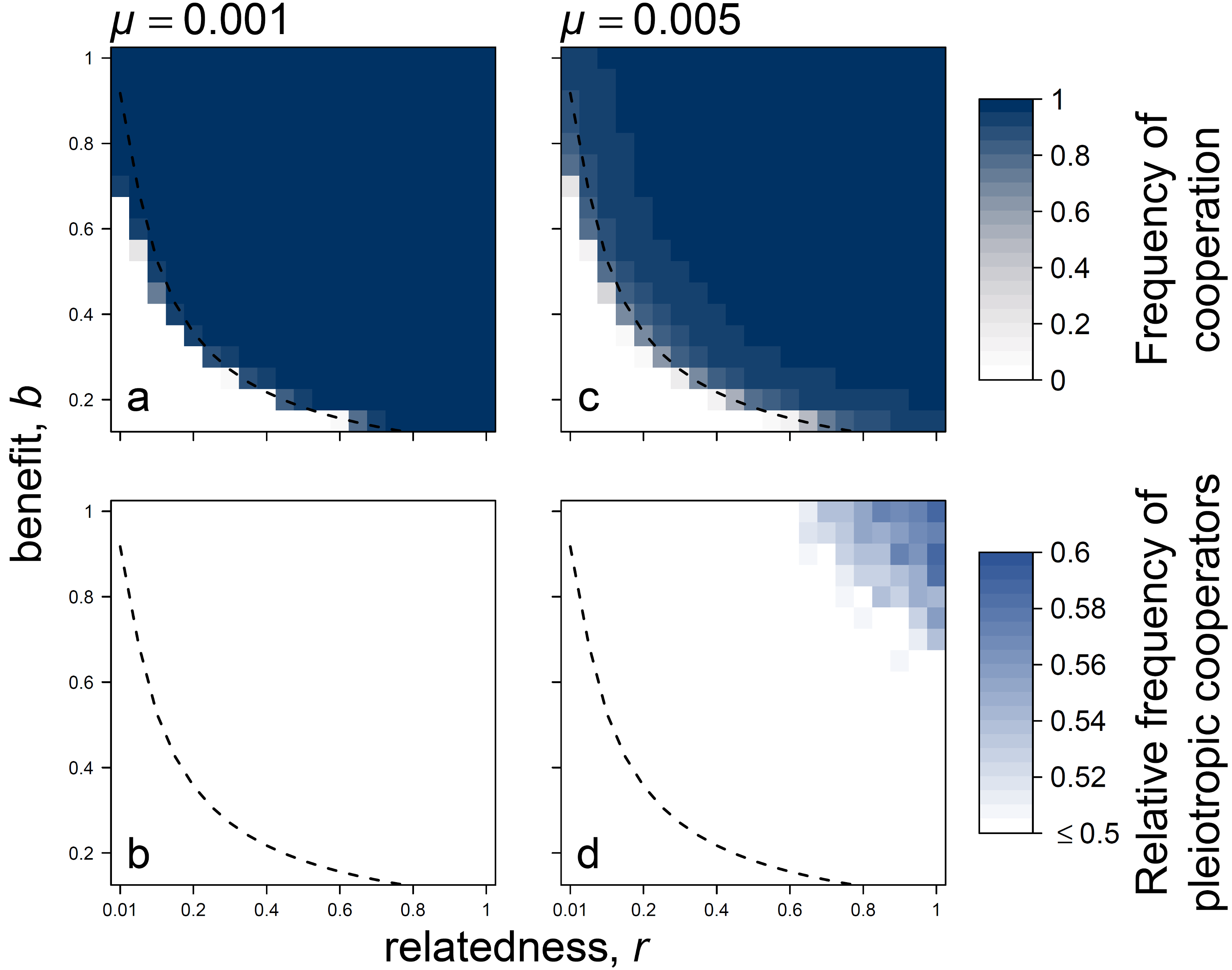

Supplement: S5 Fig — Losing a function (i.e., cooperation, private trait, and pleiotropy) is 10 times more likely than gaining one. For example, if a mutation occurs in a nonpleiotropic cooperator, it has 10 times more chances to lead to a loss of either cooperation or private production than a gain of the pleiotropic link. As before, cooperation only evolves when Hamilton’s rule is satisfied. However, pleiotropy is only favoured under high relatedness and large cooperation benefits and a high mutation rate. Panels (a) and (c) show the frequency of cooperation, and panels (b) and (d) show their respective proportion of pleiotropic cooperators. The dashed line represents the analytical prediction for when Hamilton’s rule is satisfied, assuming that migration occurs every generation (i.e., k = 1 in Eq 3 in the main text). In all panels, all genotypes and mutations I and II in Fig 3 of the main text are allowed. Parameters: c = 0.1, g = 0.5, k = 10. (TIF) [file pbio.2006671.s008.tif]

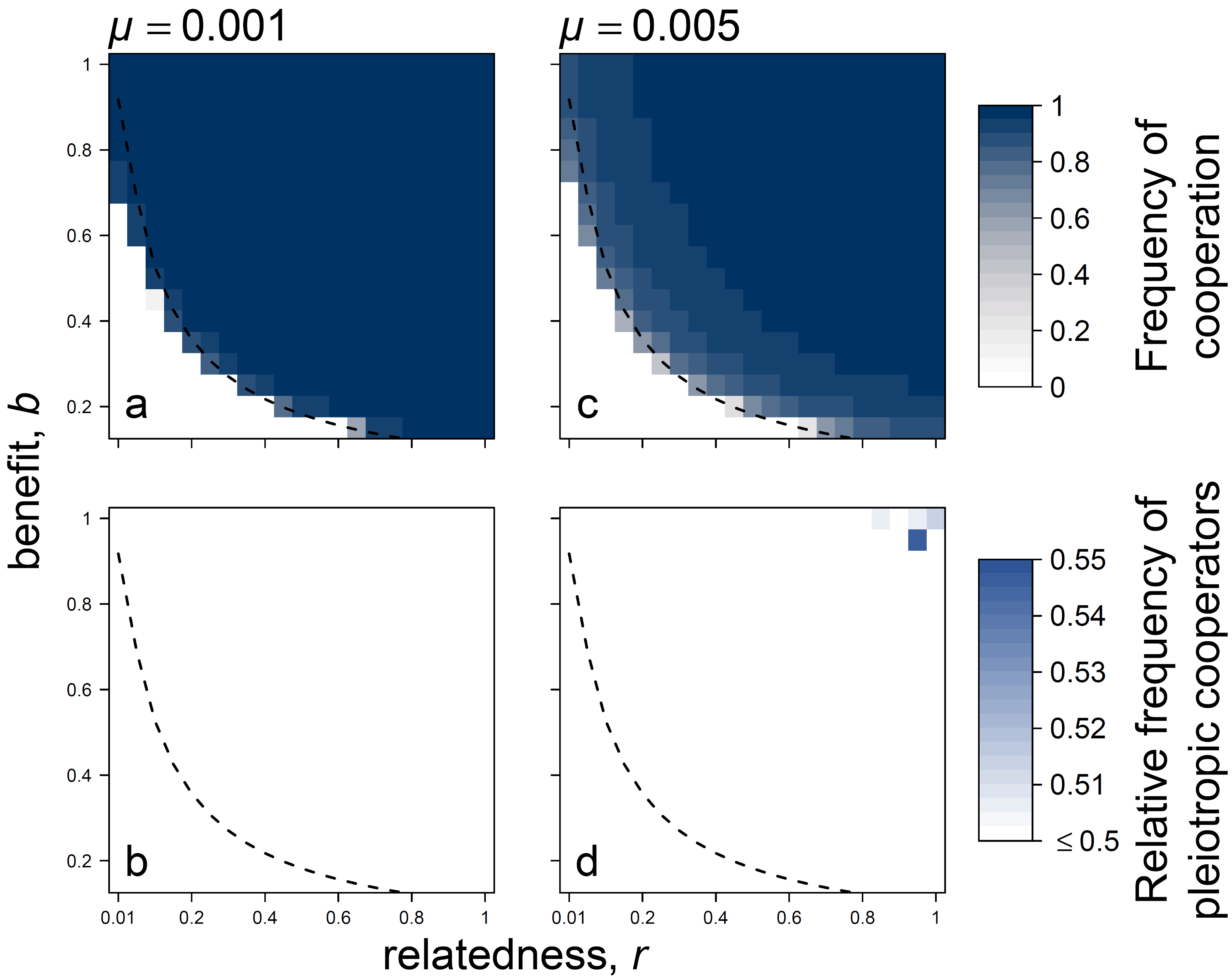

Supplement: S6 Fig — Losing a function (i.e., cooperation, private trait, and pleiotropy) is 100 times more likely than gaining one. For example, if a mutation occurs in a nonpleiotropic cooperator, it has 100 times more chances to lead to a loss of either cooperation or private production than a gain of the pleiotropic link. As before, cooperation only evolves when Hamilton’s rule is satisfied. However, pleiotropy is only favoured with relatedness and cooperation benefits close to 1 and a high mutation rate. Panels (a) and (c) show the frequency of cooperation, and panels (b) and (d) show their respective proportion of pleiotropic cooperators. The dashed line represents the analytical prediction for when Hamilton’s rule is satisfied, assuming that migration occurs every generation (i.e., k = 1 in Eq 3 in the main text). In all panels, all genotypes and mutations I and II in Fig 3 in the main text are allowed. Parameters: c = 0.1, g = 0.5, k = 10. (TIF) [file pbio.2006671.s009.tif]

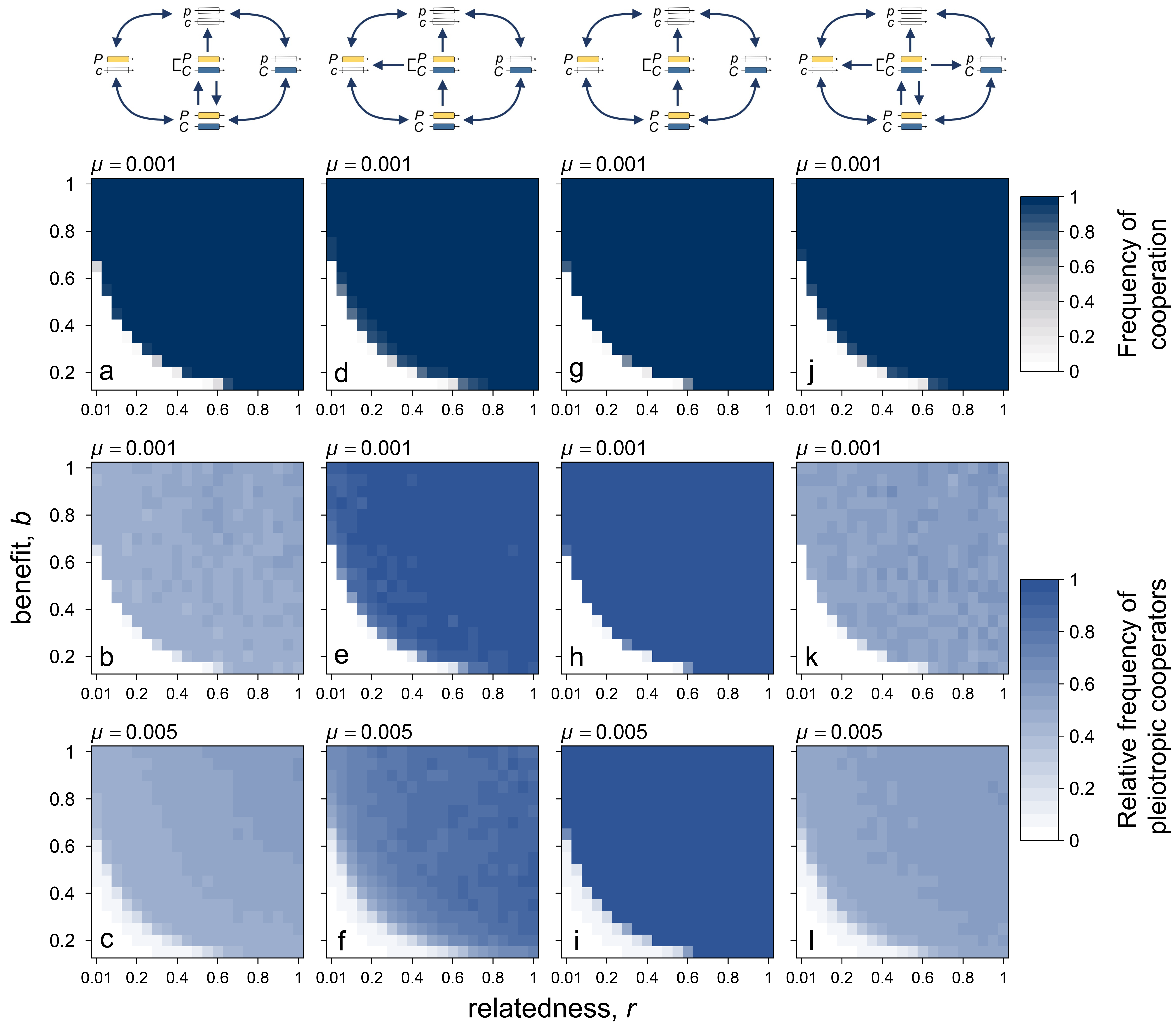

Supplement: S7 Fig — Each column represents a distinct scenario, whose corresponding genetic architecture is shown at the top. In (a-c) and (j-l), pleiotropy can revert to nonpleiotropic cooperation (mutation II in Fig 3 in the main text). In (d-f) and (j-l), mutations on the cooperation trait in pleiotropic individuals are possible and generate cheats. In (j-l), mutations on the private trait in pleiotropic individuals are possible. Pleiotropy prevails only when Hamilton’s rule is satisfied when in competition with all nonpleiotropic genotypes (Fig 3 in the main text). Whenever pleiotropy cannot revert to a two-regulator system (panels d-i), the population is entirely invaded by pleiotropic cooperators. Parameters: c = 0.1, g = 0.5. (TIF) [file pbio.2006671.s010.tif]

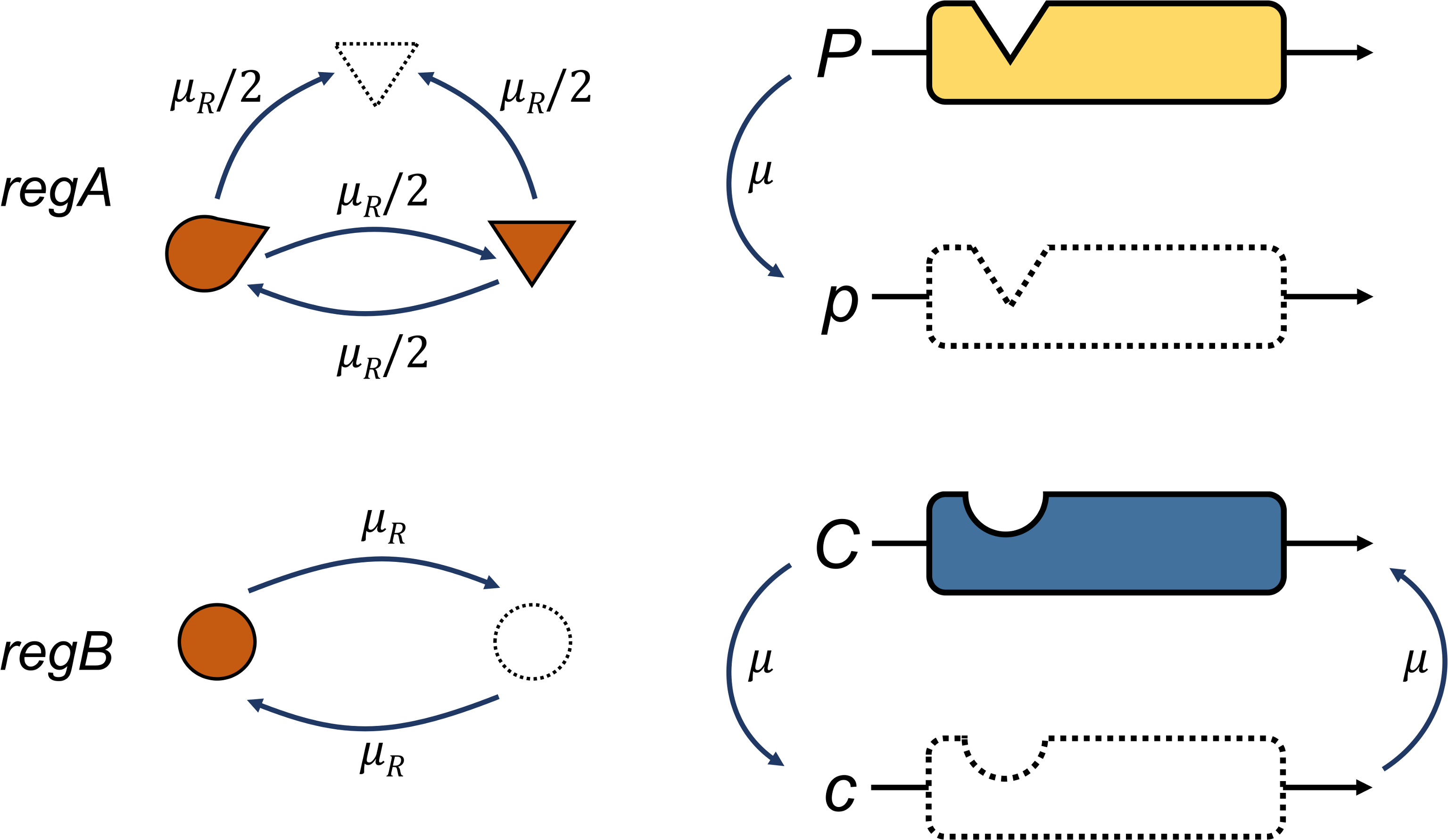

Supplement: S8 Fig — We model more explicitly the pleiotropic link between the private and cooperation genes. Each gene is expressed only if both the expressing version of the allele (C or P) and the corresponding expressing version of its regulator (filled regA for the private trait and filled regB for the cooperation trait) are present. We assume that each regulator can be lost and that the private regulator, regA, can become pleiotropic by being able to regulate both genes at the same time (pointy orange circle). During reproduction, each regulator mutates independently with probability μR, and each gene mutates with probability μ. We assume that the private trait is essential, so in case the private regulator and/or private gene is lost through mutation, the individual dies. All the possible genotypes resulting from these elements and their corresponding phenotypes are listed in S2 Table. (TIF) [file pbio.2006671.s011.tif]

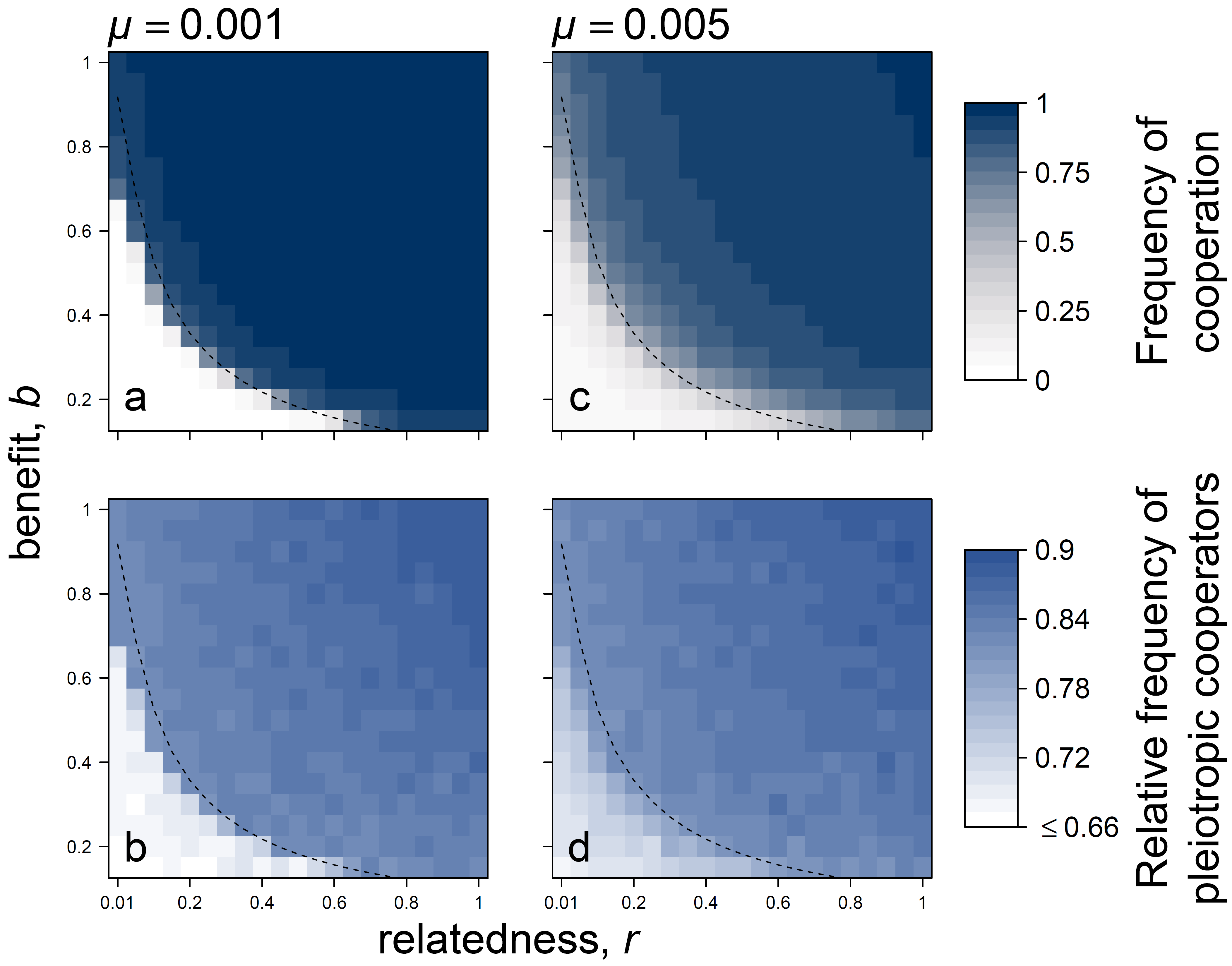

Supplement: S9 Fig — Panels (a) and (c) show the frequency of cooperation, and panels (b) and (d) show their respective relative proportion of pleiotropic cooperators (i.e., frequency of genotype number g20 + g24 over the sum of frequencies of the genotype number g16, g20, g24). The dashed line represents the analytical prediction r(N − 1)b/N = c − b/N, for when Hamilton’s rule is satisfied, assuming that migration occurs every generation (i.e., k = 1). Parameters: np = 500, c = 0.1, g = 0.5, k = 10, μR = 0.001. (TIF) [file pbio.2006671.s012.tif]

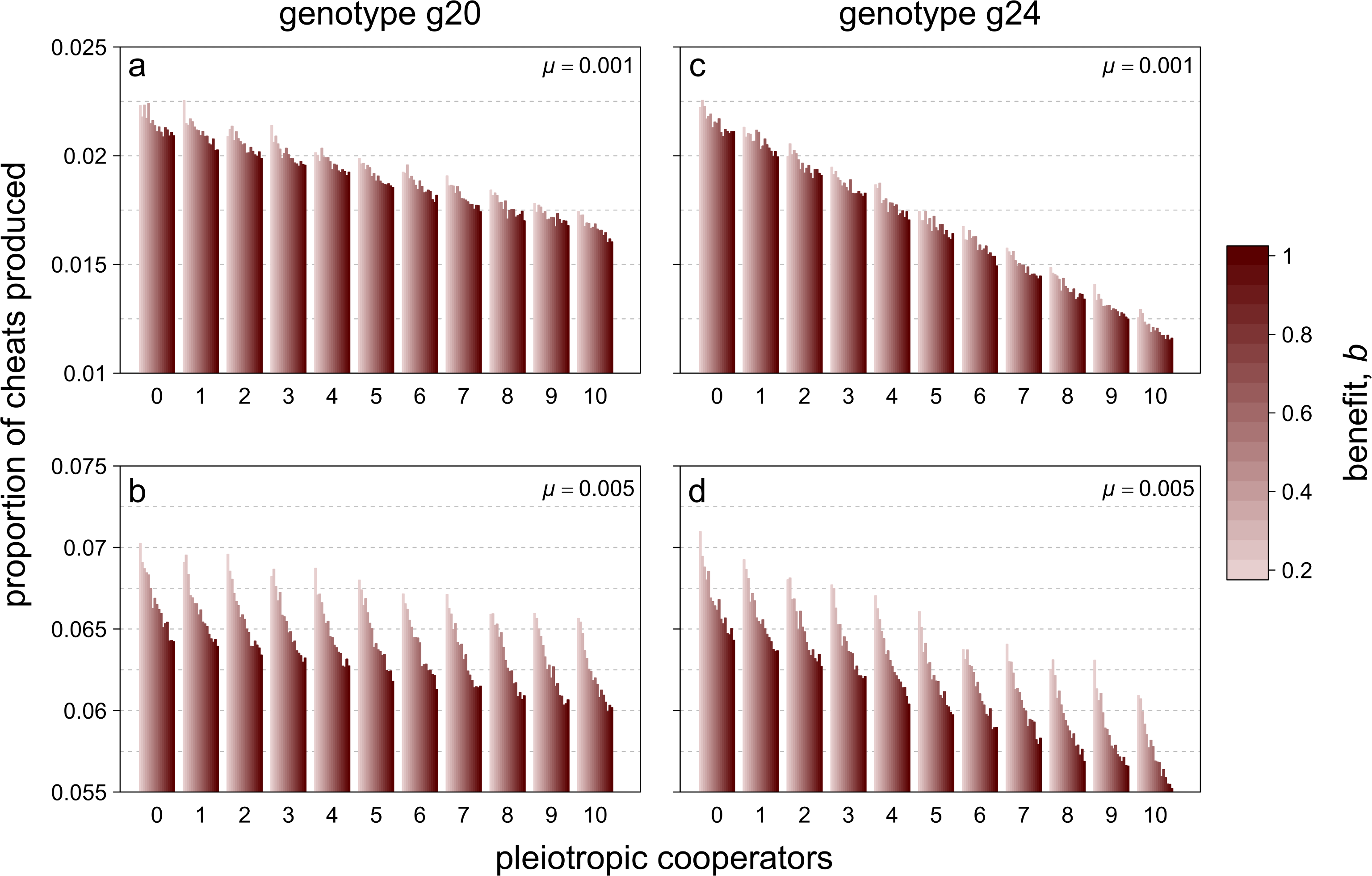

Supplement: S10 Fig — All panels show the proportion of cheats produced during a single growth phase for different values of the cooperation benefit b and mutation rate μ. Each patch is started with 10 cooperators, and the x axis shows the number of those cooperators for which cooperation was pleiotropically linked to an essential private trait, using pleiotropic cooperators with genotype number g20 (a-b) or g24 (c-d). Increased pleiotropy leads to a decreased accumulation of cheats. Each bar represents the average of 104 patches. Parameters: c = 0.1, g = 0.5, k = 10, μR = 0.001. (TIF) [file pbio.2006671.s013.tif]

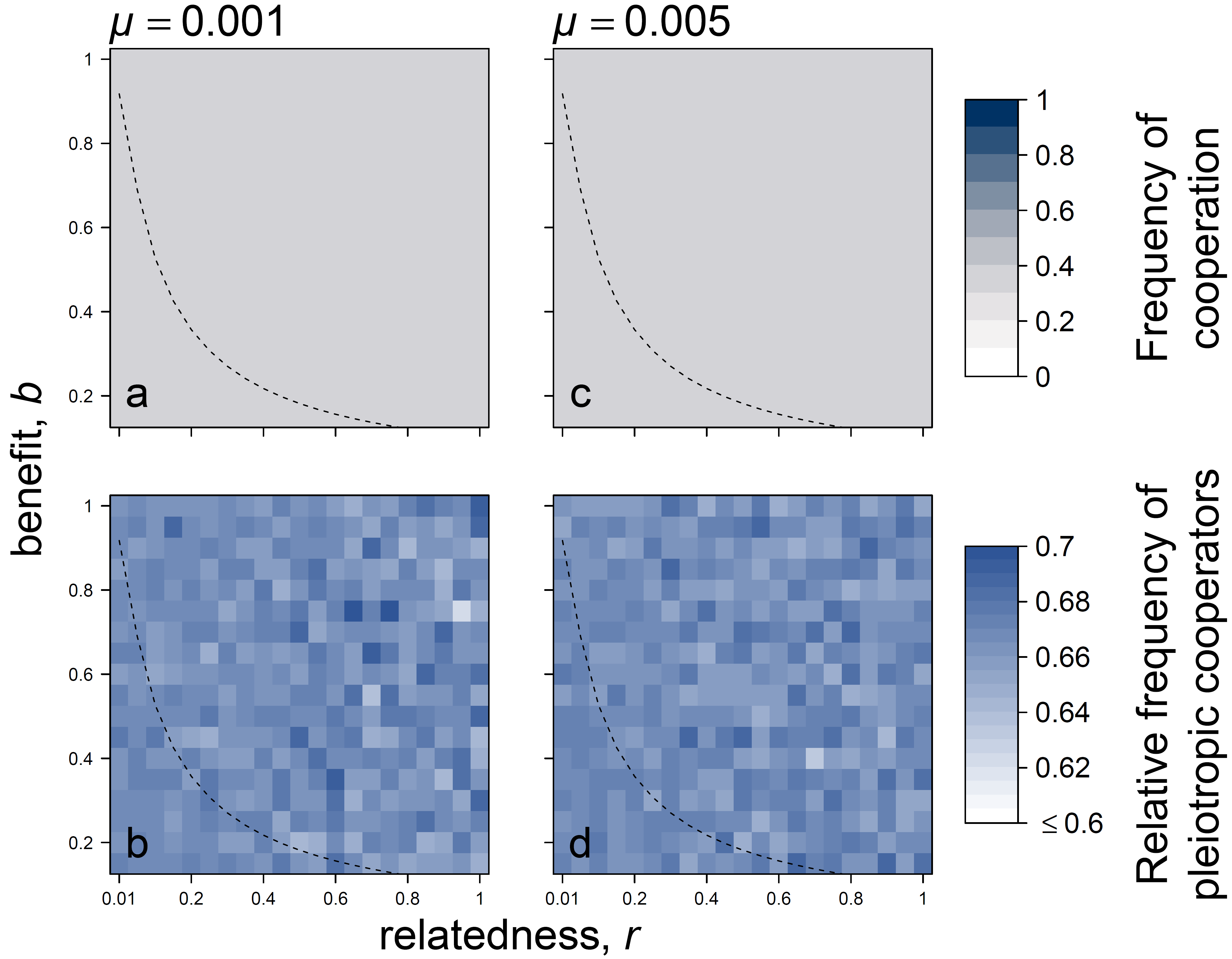

Supplement: S11 Fig — Panels (a) and (c) show the frequency of cooperation, which ranges between 0.36 and 0.38. This is because there are three cooperative genotypes among a total of 8 viable genotypes (S2 Table), with 3/8 = 0.375. Panels (b) and (d) show their respective relative proportion of pleiotropic cooperators (i.e., frequency of genotype number g20 + g24 over the sum of frequencies of the genotype number g16, g20, g24). The dashed line represents the analytical prediction r(N − 1)b/N = c − b/N, for when Hamilton’s rule is satisfied, assuming that migration occurs every generation (i.e., k = 1). All runs are neutral with respect to the cooperation trait but not the essential private trait; i.e., all individuals that express the essential private trait have fitness 1 + g, whereas the others die. Parameters: np = 500, g = 0.5, k = 10, μR = 0.001. (TIF) [file pbio.2006671.s014.tif]

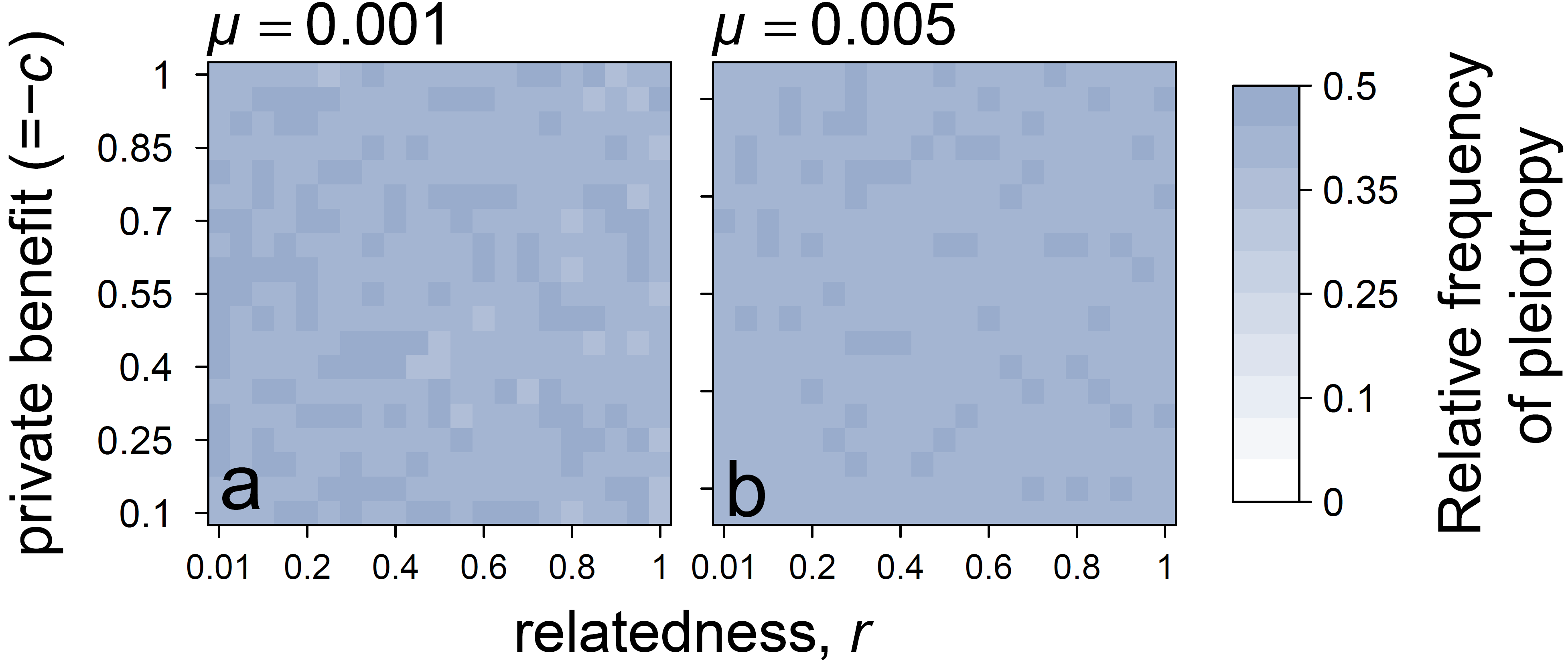

Supplement: S12 Fig — As in our baseline scenario, the private trait is essential. However, the cooperation trait is replaced by a second privately beneficial trait (with b = 0 and c < 0). As a result, individuals expressing both private traits can either be pleiotropic or nonpleiotropic. In both panels, individuals with a pleiotropic link between two private traits are never more common than individuals without this pleiotropic link, as their proportion never exceeds 50%. Parameters: b = 0, g = 0.5, k = 10. (TIF) [file pbio.2006671.s015.tif]

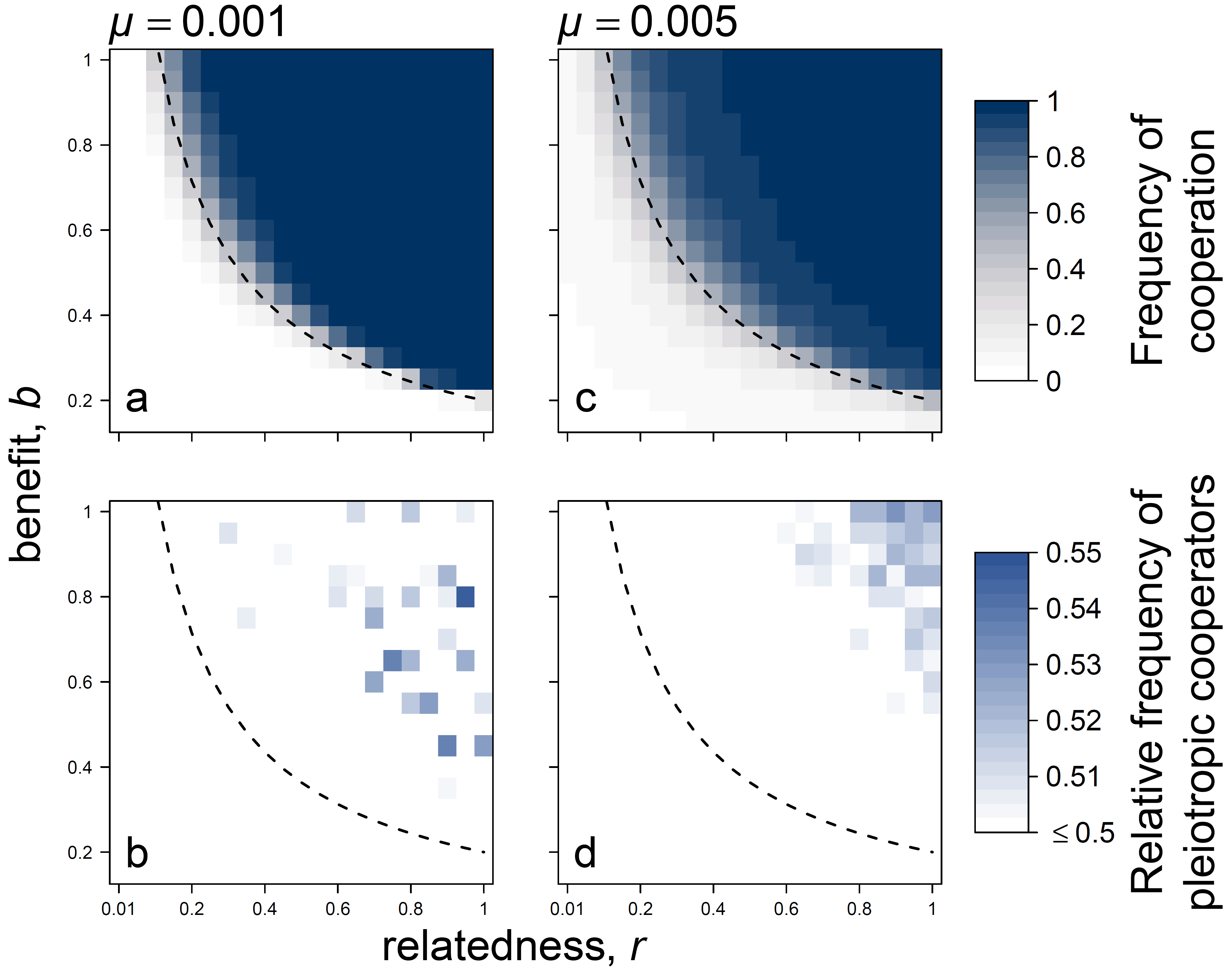

Supplement: S13 Fig — The benefit of cooperation is alternating between 0 and the value shown on the y axis every 10 generations (i.e., every growth phase). Cooperation is less likely to evolve under such fluctuating environment, and pleiotropy only evolves when Hamilton’s rule is satisfied. Panels (a) and (c) show the frequency of cooperation, and panels (b) and (d) show their respective proportion of pleiotropic cooperators. The dashed line represents the analytical prediction for when Hamilton’s rule is satisfied, assuming that the benefit is b/2 and that migration occurs every generation (i.e., k = 1; and substituting b = b/2 in Eq 3 in the main text). In all panels, all genotypes and mutations I and II in Fig 3 of the main text are allowed. Parameters: c = 0.1, g = 0.5, k = 10. (TIF) [file pbio.2006671.s016.tif]

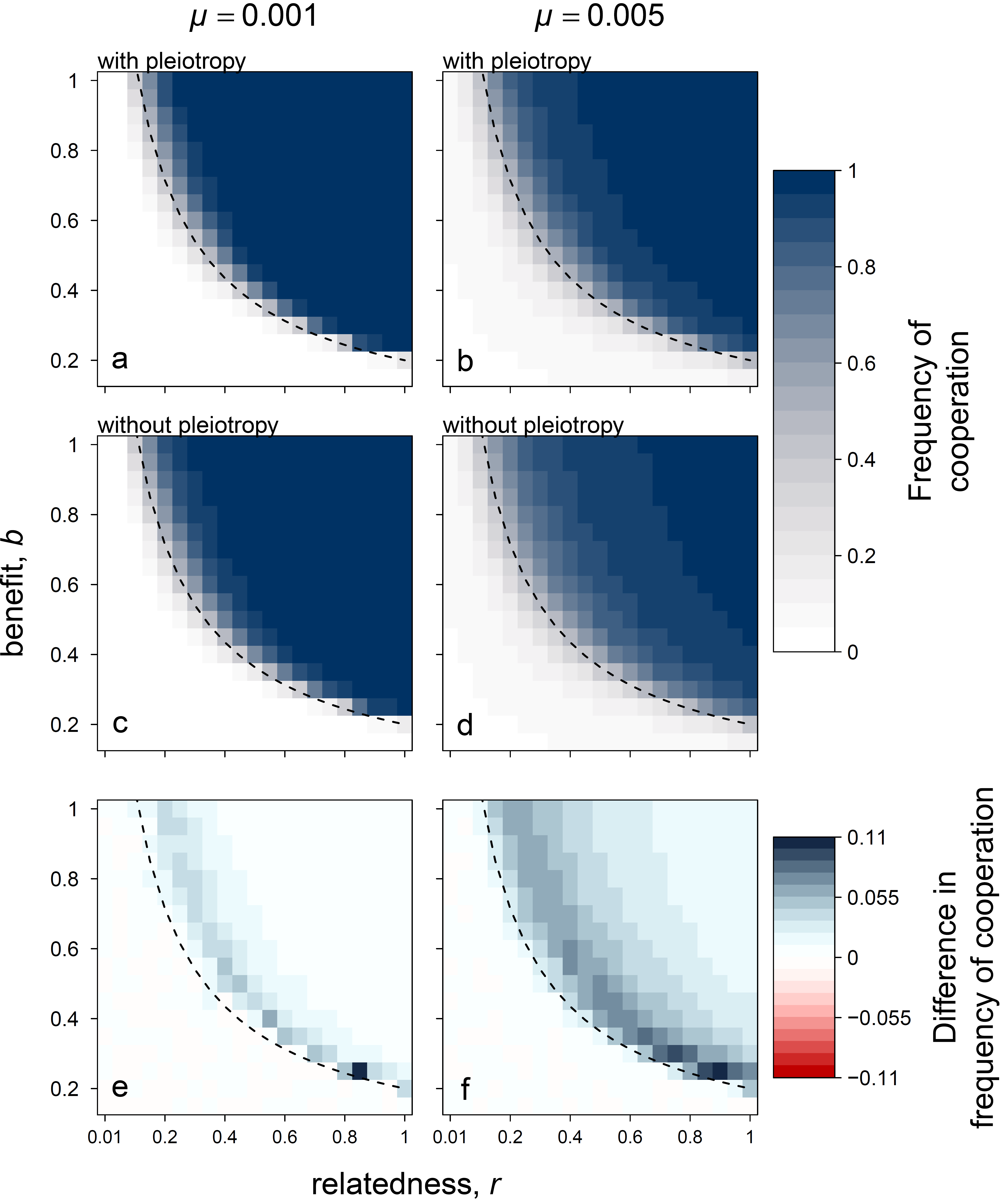

Supplement: S14 Fig — The benefit of cooperation is alternating between 0 and the value shown on the y axis every 10 generations (i.e., every growth phase). Cooperation is more likely to evolve if pleiotropy is allowed. In (a) and (b), pleiotropic cooperators are allowed (all genotypes and mutations I and II in Fig 3 in the main text). In (c) and (d), pleiotropic cooperators are replaced by nonpleiotropic cooperators (this maintains a similar ratio of cooperative strategies to when pleiotropic cooperators are present). Panels (e) and (f) show the difference in cooperation frequency between (a) and (c) and (b) and (d), respectively. The dashed line represents the analytical prediction for when Hamilton’s rule is satisfied, assuming that the benefit is b/2 and that migration occurs every generation (i.e., k = 1; and substituting b = b/2 in Eq 3 in the main text). Parameters: c = 0.1, g = 0.5, k = 10. (TIF) [file pbio.2006671.s017.tif]

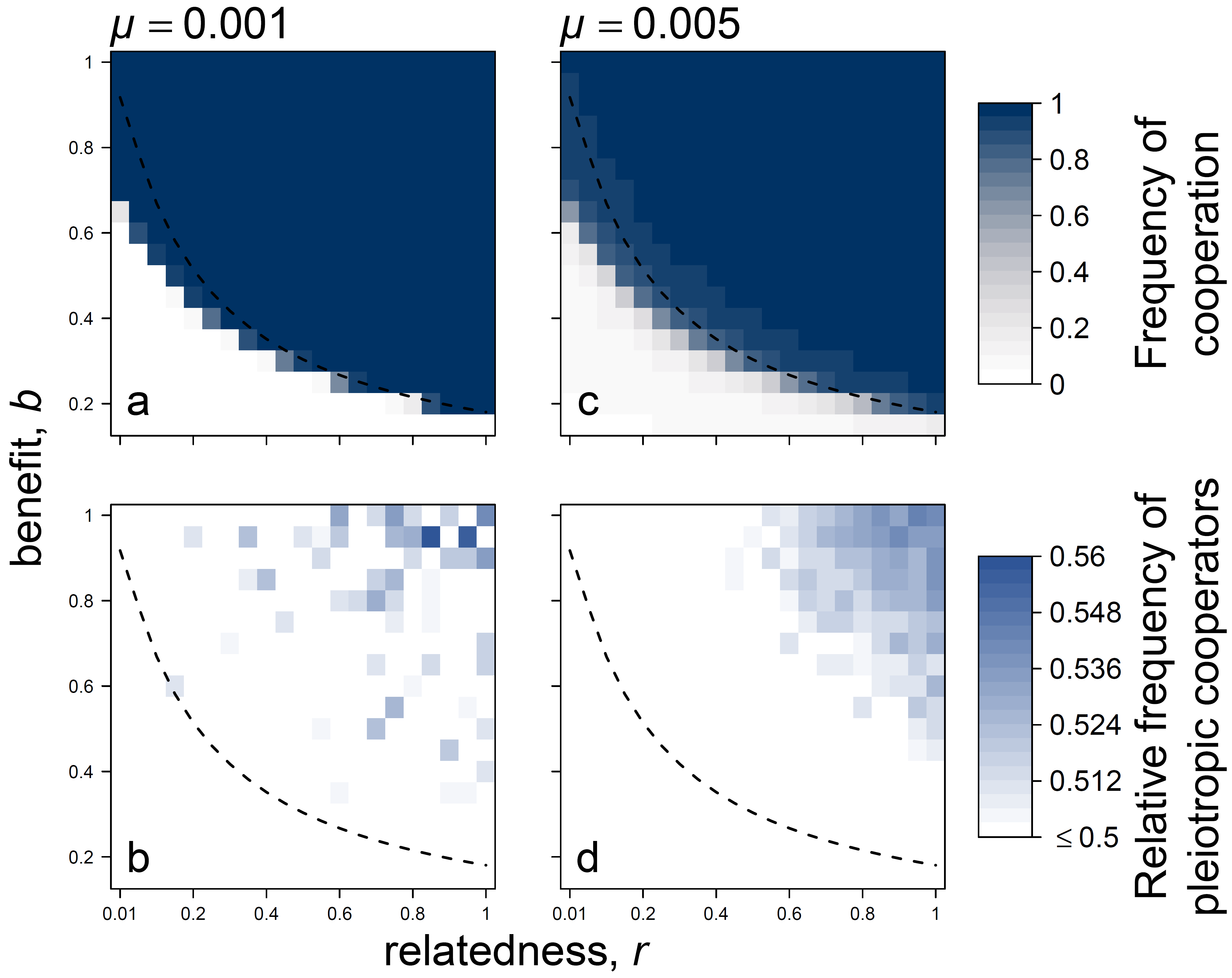

Supplement: S15 Fig — Population structure (relatedness r) is alternating between 0.01 and the value shown on the x axis every 10 generations (i.e., every growth phase). Cooperation is less likely to evolve under such fluctuating environment, and pleiotropy only evolves when Hamilton’s rule is satisfied. Panels (a) and (c) show the frequency of cooperation, and panels (b) and (d) show their respective proportion of pleiotropic cooperators. The dashed line represents the analytical prediction for when Hamilton’s rule is satisfied assuming that relatedness is (r + 0.01)/2 and that migration occurs every generation (i.e., k = 1; and substituting r = [r + 0.01)/2 in Eq 3 in the main text). In all panels, all genotypes and mutations I and II in Fig 3 of the main text are allowed. Parameters: c = 0.1, g = 0.5, k = 10. (TIF) [file pbio.2006671.s018.tif]

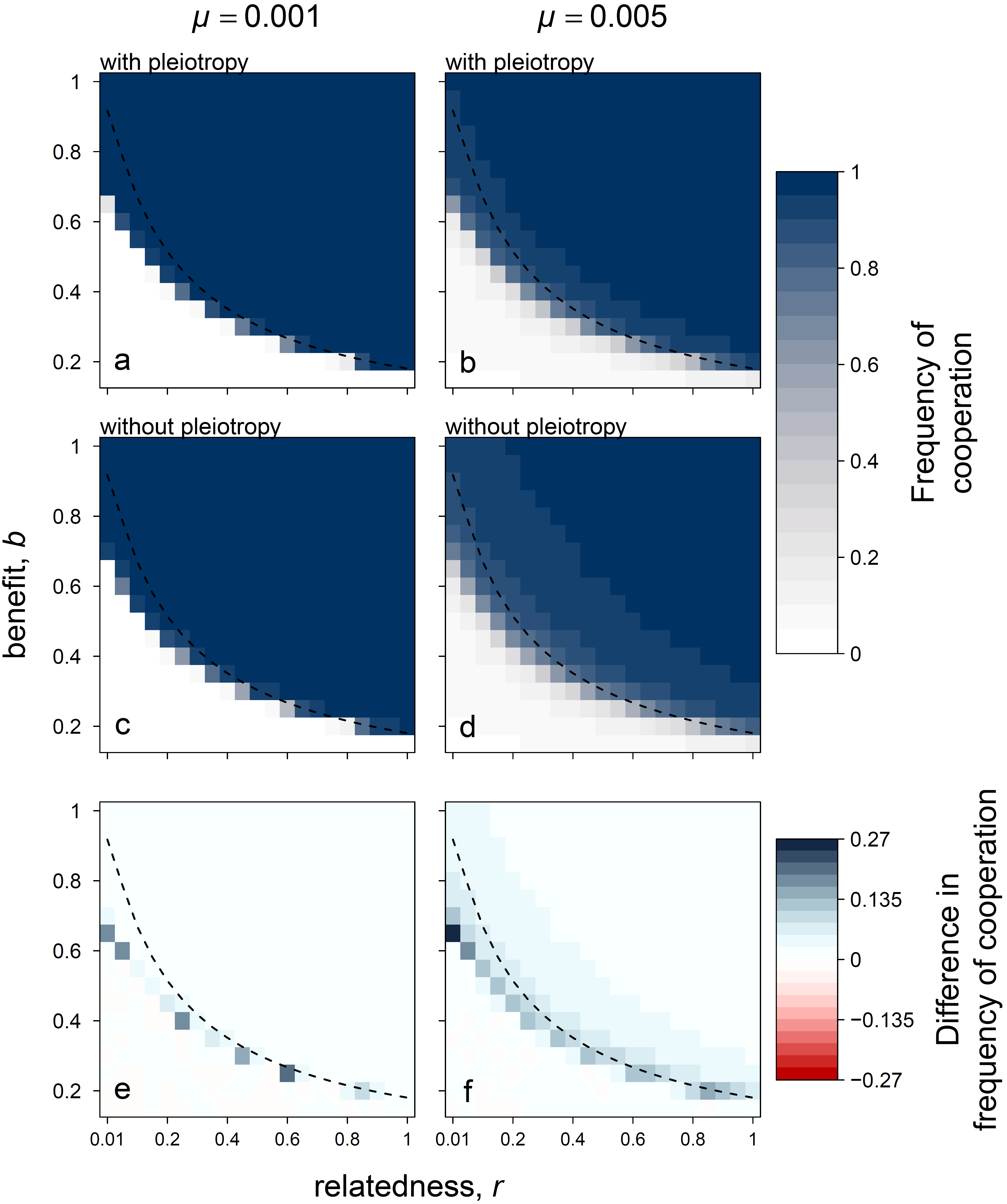

Supplement: S16 Fig — Population structure (relatedness r) is alternating between 0.01 and the value shown on the x axis every 10 generations (i.e., every growth phase). Cooperation is more likely to evolve if pleiotropy is allowed. In (a) and (b), pleiotropic cooperators are allowed (all genotypes and mutations I and II in Fig 3 of the main text). In (c) and (d), pleiotropic cooperators are replaced by nonpleiotropic cooperators (this maintains a similar ratio of cooperative strategies to when pleiotropic cooperators are present). Panels (e) and (f) show the difference in cooperation frequency between (a) and (c) and (b) and (d), respectively. The dashed line represents the analytical prediction for when Hamilton’s rule is satisfied assuming that relatedness is (r + 0.01)/2 and that migration occurs every generation (i.e., k = 1; and substituting r = [r + 0.01]/2 in Eq 3 in the main text). Parameters: c = 0.1, g = 0.5, k = 10. (TIF) [file pbio.2006671.s019.tif]

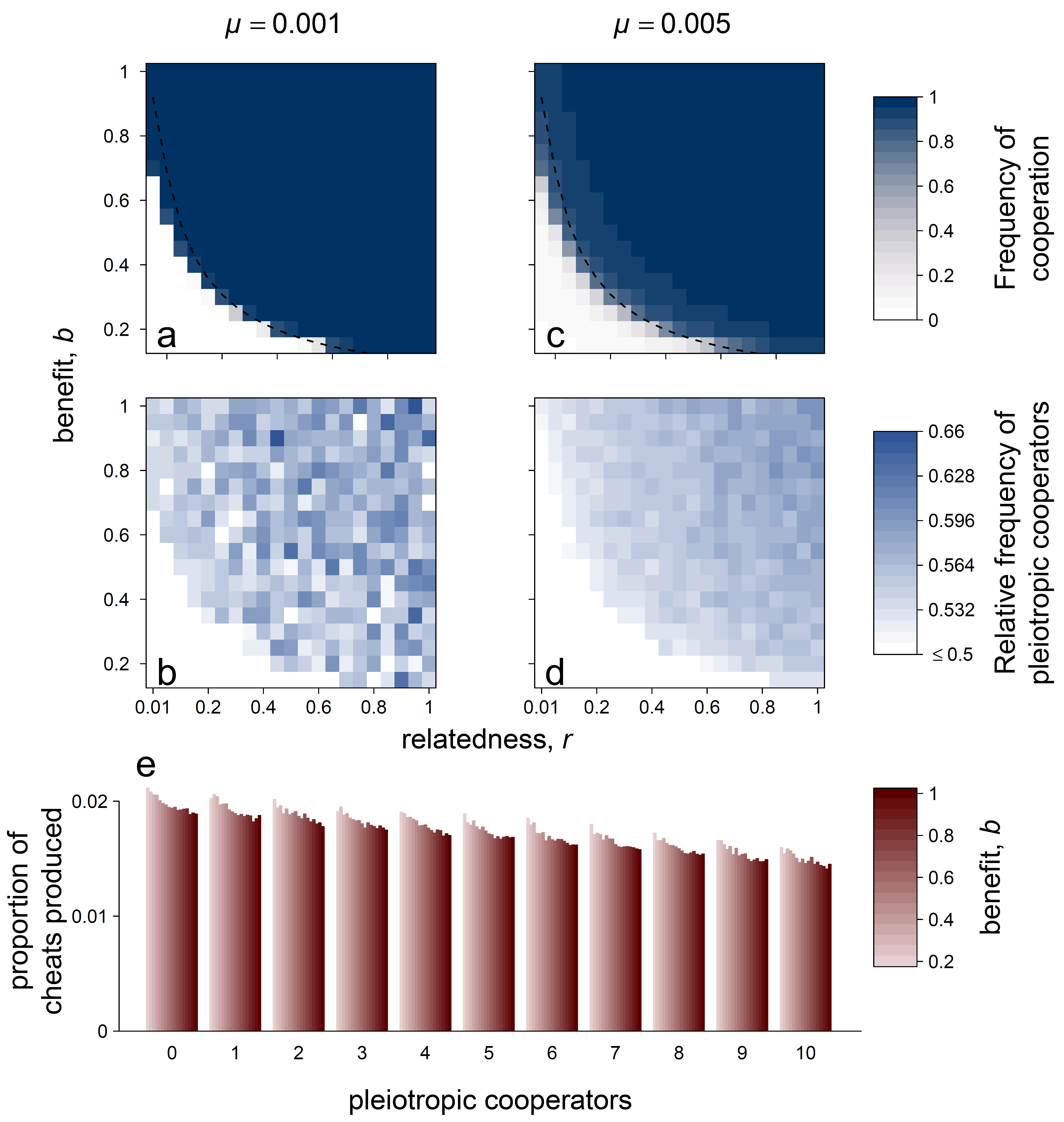

Supplement: S17 Fig — Panels (a) and (c) show the frequency of cooperation for different mutation rates μ, and panels (b) and (d) show their respective proportion of pleiotropy relative to all cooperative genotypes (i.e., pleiotropic cooperators, nonpleiotropic cooperators, and cooperative private nonproducers). The dashed lines represent the analytical prediction for when Hamilton’s rule is satisfied assuming that migration occurs every generation (i.e., k = 1; Eq 3 in the main text). Panel (e) shows the proportion of cheats produced by mutation and growth during a single growth phase (mutation rate μ = 0.005) for different values of the cooperation benefit b. Each patch is started with 10 cooperators, and the x axis shows the number of those cooperators for which cooperation was pleiotropically linked to an essential private trait. Increased pleiotropy leads to a decreased accumulation of cheats, but to a lesser extent than when mutations in pleiotropic individuals cannot generate cheats (compare panel [c] with Fig 4C in the main text). Each bar represents the average of 104 patches. Parameters: c = 0.1, g = 0.5, k = 10. (TIF) [file pbio.2006671.s020.tif]
